# Electro-Encephalography and Electro-Oculography in Aeronautics: A Review Over the Last Decade (2010–2020)

**DOI:** 10.3389/fnrgo.2020.606719

**Published:** 2020-12-21

**Authors:** Chama Belkhiria, Vsevolod Peysakhovich

**Affiliations:** ISAE-SUPAERO, Université de Toulouse, Toulouse, France

**Keywords:** human factors, aeronautics, cognition, brain computer interface, signal processing, mental workload, fatigue

## Abstract

Electro-encephalography (EEG) and electro-oculography (EOG) are methods of electrophysiological monitoring that have potentially fruitful applications in neuroscience, clinical exploration, the aeronautical industry, and other sectors. These methods are often the most straightforward way of evaluating brain oscillations and eye movements, as they use standard laboratory or mobile techniques. This review describes the potential of EEG and EOG systems and the application of these methods in aeronautics. For example, EEG and EOG signals can be used to design brain-computer interfaces (BCI) and to interpret brain activity, such as monitoring the mental state of a pilot in determining their workload. The main objectives of this review are to, (i) offer an in-depth review of literature on the basics of EEG and EOG and their application in aeronautics; (ii) to explore the methodology and trends of research in combined EEG-EOG studies over the last decade; and (iii) to provide methodological guidelines for beginners and experts when applying these methods in environments outside the laboratory, with a particular focus on human factors and aeronautics. The study used databases from scientific, clinical, and neural engineering fields. The review first introduces the characteristics and the application of both EEG and EOG in aeronautics, undertaking a large review of relevant literature, from early to more recent studies. We then built a novel taxonomy model that includes 150 combined EEG-EOG papers published in peer-reviewed scientific journals and conferences from January 2010 to March 2020. Several data elements were reviewed for each study (e.g., pre-processing, extracted features and performance metrics), which were then examined to uncover trends in aeronautics and summarize interesting methods from this important body of literature. Finally, the review considers the advantages and limitations of these methods as well as future challenges.

## Introduction

Electro-encephalography (EEG) and electro-oculography (EOG) are methods of electrophysiological monitoring in neuroscience and clinical exploration. EEG and EOG signals can be used in the design of brain-computer interfaces (BCI) that interpret brain activity. Due to the fact that they are straightforward approaches to evaluating brain oscillations and eye movements, and because they use standard laboratory and/or mobile techniques, EEG and EOG have in recent years been applied to the aeronautical industry.

This review describes the potential of these systems when applied in aeronautics. The main objectives are, (i) to offer an in-depth review of literature on the basics of EEG and EOG and their application in aeronautics; (ii) to explore the methodology and trends of research in combined EEG-EOG studies over the last decade; and (iii) to provide methodological guidelines for beginners and experts when applying these methods in environments outside the laboratory, with a particular focus on human factors and aeronautics.

The review is structured as follows: section Introduction first describe EEG and EOG techniques, the main approaches to acquiring signals, and the use of them in aeronautics, before concluding with discussions of the motivations for applying them to aeronautics, and a discussion of contributions to this field. Section Methodology of the Review describes the methodology used to construct the review and taxonomy table, respectively. Section Results then presents the results of the review, including the trend analyses. Finally, section Discussion discusses research over the last decade with a focus on combined approaches to EEG-EOG and the relevance of this approach to aeronautics.

### Electroencephalography (EEG)

#### Origin of EEG Response

EEG is one of the most important methods of evaluating brain disorders and monitoring the electrical behavior of the brain. The EEG also has the major advantage of excellent temporal resolution, which enables it to study neural activity at a millisecond scale, and best approximates the neural timing. EEG allows for the analysis of the various rhythms generated by different cortical regions. The current produced by the electrical activity of neurons reaches the surface of the scalp. EEG offers a non-invasive method of recording the difference between the potentials that are generated by neural sources and annoying non-neural artifacts. As the signal induces important temporal and spatial variations, the electrode positions are determined using multiple channels settled by the international 10–20 standard. Recommendations for the use of EEG equipment in assessments are provided by the International Federation of Clinical Neurophysiology (Babiloni et al., 2020). The electrical signal is diffused from electrodes placed on the scalp to an external amplifier that intensifies the potentials. EEG signals are commonly detected between 20 and 150 μV in the 0.5–60 Hz band (Binnie et al., 1982). The signal is constantly sampled to provide a relevant temporal resolution to explore event-related potentials (ERP) and the EEG power spectrum. In research focused on frequency-based analyses (such as prefrontal lateralization of alpha or beta bands), a sampling rate of 128 Hz can be sufficient. When the objective is high time precision measurements (such as language-related high gamma activity), the EEG should ideally collect data at a high sampling rate (>500 Hz).

EEG analysis could be conducted in the time domain, frequency domain, or time-frequency domain. Extracting temporal features (e.g., amplitude, power, average periodicity, and synchronization) provides useful qualitative information for the classification. However, all of these temporal characteristics do not describe the signal in its integrity. During a seizure, for instance, the signal is not stable, and it is necessary to separate the frequency components to classify the seizure. Therefore, in addition to time-domain features, frequency domain exploration is also needed to detect and classify all types of seizures. A time-domain analysis provides better spatial information, thus poor frequency content information is required for EEG classification. The frequency-domain can provide time information when the function is windowed. The choice of window size is the biggest challenge in frequency analysis. Time-frequency analysis solves these two problems. Some EEG investigations consider that wavelet analysis is the best method for time-frequency analysis. Generally, authors apply a series of transformations e.g., Fourier transform (Radha et al., 2014), Short Time Fourier Transform (Görür et al., 2003), Wavelet transform (Fraiwan et al., 2012), Hilbert-Hung transform (Li et al., 2009), and Empirical Mode Decomposition (Hassan and Bhuiyan, 2016). Spectral analyses, based on Fourier Transform, are then commonly used to convert the time function into different frequencies and to calculate the amplitude in each frequency band. The frequency bands are universally classified as the following: slow and sleep wave delta (2–4 Hz), arousal wave theta (4–8 Hz), relaxation wave alpha (8–12 Hz), and active wave beta (13–32 Hz). Sensorimotor rhythm frequency bands (13–15 Hz) are related to the sensorimotor rhythm and entitled as low beta. Delta waves are commonly frontally located in adults and posteriorly in children. Theta waves are mainly recorded in frontal areas during low brain activities, sleep, or drowsiness and cognitive processing. Alpha waves are among the first rhythmic waves documented and are recorded during relaxed conditions at decreased attention levels and in a wakeful state. The alpha waves are located in the occipital area and can be induced by closing eyes. Beta waves are often recorded in frontal or central areas when the eyes are open and are related to consciousness, alertness, arousal, and motor behaviors (Barry and De Blasio, 2017). Cognitive processes such as attention, learning, and diverse types of memory occur during gamma frequencies (over 33 Hz). Unconventional classifications have also been analyzed in some studies (Caldwell et al., 2002; Gevins et al., 2003; Dahlstrom and Nahlinder, 2009; Holm et al., 2009; De Vico Fallani et al., 2012; Zhang et al., 2019a). It is worth mentioning that the frequency limits of specific waves are conventional, as there is no proper way of determining their exact values.

Many studies on oscillations in brain dynamics have indicated that during fatigue accumulation and sustained attention, increased EEG power is detected in theta frequency bands in the frontal, parietal, and central regions. Theta power increase has been detected during working memory load situations (Klimesch et al., 2007), visual tasks (Yamada, 1998), flight simulations (Smith-Jentsch et al., 2001; Dussault et al., 2004; Borghini et al., 2014), and air-traffic control simulations (Postma et al., 2005). A decrease in the alpha is known to occur during complex and cognitively demanding tasks. It has also been shown (Postma et al., 2005) that alpha and beta bands are different between the beginning and the end of a mental fatigue task. Increases in theta and decreases in alpha oscillations have also been associated with an increase in the accuracy of task performance (Klimesch et al., 1999). Interestingly, such an increase in EEG power in the theta band can be used to characterize a single task from a multi-task activity performed by pilots (Borghini et al., 2014). Varied EEG approach montages are given in complex higher-order cognitive operations.

#### EEG Approaches

Although great importance has been placed on classic unmovable EEG montages, they have disadvantages in that they are much bulkier and more time and effort consuming than the new generation of wearable EEG. The concept of wearable EEG improves upon the bulky and limited mobility of classic montages, using small devices that can record EEG outside of laboratory conditions. These miniaturized devices have the advantage of being able to detect EEG signals for short or long periods, for example when sleeping, which significantly improves the brain-computer interface (BCI) monitoring method. The importance of using the wireless EEG in BCI monitoring is to maximize wearability, enabling unconstrained mobility, usability, and reliability in operational environments.

As well as involving long preparation time and bulky design, classical scalp-mounted EEG are not suited to situations that involve environmental artifacts (e.g., aviation and space operation, or patients with a cochlear implant or hearing aid; Nogueira et al., 2019). To address these issues, novel EEG technologies use tiny electrodes that are placed externally around the ear (Debener et al., 2015; Bleichner and Debener, 2017) or involve in-ear electrodes (Looney et al., 2011). The cEEGrid electrode array (Debener et al., 2015) is a promising device with 10 electrodes printed onto a C-shaped flexible board, enabling it to fit around and measure EEG data behind the ear. It also uses a small amount of electrolyte electrode gel, which ensures low-impedance contact between the cEEGrid electrodes and the skin. The capacity is stable because the gel does not dry-up. The cEEGrid electrode array is then connected to a micro wireless amplifier and uses signal recordings from a cellphone. This accessible design means that it has potential applications in clinical settings, aeronautics, and other research areas.

With a reduced number of electrodes, the difference between cEEGrid performance and scalp EEG performance can be explained by the position of the reference electrode. The cEEGrid uses a local reference, with ground and recording electrodes allocated around the ear, giving out small signal amplitudes. However, for a scalp EEG with a smaller number of electrodes around the ear, the tip of the nose is used as a reference, and its position is farther from the recording electrode, and therefore gives higher amplitudes. In line with conventional EEG results, Debener et al. (2015) identified ERPs and alpha frequencies during an auditory oddball task with open eyes using the cEEGrid. Advanced explorations proved that the cEEGrid can detect neural signals to select the voice of a speaker with high precision, and can be even used as a BCI monitor for hearing aids (Mirkovic et al., 2016). Recently, the cEEGrid was successfully used in decoding selective attention in normal hearing and cochlear implant patients (Nogueira et al., 2019). Looney et al. (2011) presented the in-ear EEG device. It records to the same standards as the conventional scalp electrodes. However, the device relies on custom-made hearing aid earplugs, which take a wax impression of the ears (outer ear and external ear canal). The important benefits of in-ear EEG includes easy set-up; time of installation; accessibility for people with hair that is incompatible with EEG recordings; and durability because they are fixed in the ear canal. They are comfortable to wear, discreet, resembling earphones, earbuds, and earplugs, and facilitating everyday use. Contrary to scalp EEG, the in-ear EEG devices are easy to place without the presence of experienced assistants. They are held firmly in place and thus diminish motion artifacts. Since the electrodes are fixed on the earpiece surface, they offer a precise spatial positioning which decreases the inter-experiment variability. The feasibility of in-ear EEG for cognitive assessment has been studied in a few exploratory papers (Stochholm et al., 2016; Von Rosenberg et al., 2016; Zibrandtsen et al., 2016), which claim that in-ear EEG is a promising candidate for forthcoming explorations based on human monitoring technology (BCI, aviation, and space).

Despite advances in mobile EEG systems, around and in-ear EEG have some drawbacks. Compared to conventional scalp EEGs, these alternative systems have fewer electrodes and cover much-reduced regions. Therefore, it is recognized that its brain source analysis is less accurate than that of conventional scalp EEG. Note that physiological artifacts caused by the electrical activity of the skin are unavoidable, but may be relatively easy to deal with by temporal filtering and other post-processing procedures (Reis et al., 2014). The quality of the scalp EEG signal depends on the connection between the amplifier input and the skin surface. Wet electrodes based on conductive gel guarantee low impedance levels (<10 kΩ). Given that dry electrodes are placed on the skin without any gel application, the dry EEG system typically results in larger impedance than wet systems (Brown et al., 2010; Chen et al., 2014). To date, few studies have directly compared the data quality between these two systems. A recent study (Hinrichs et al., 2020) found that the resting state EEG power and ERP were comparable between the two systems. Di Flumeri et al. (2019) evaluated three different dry electrode types when compared with wet electrodes in terms of signal spectral features, mental state classification, and usability. The dry electrodes included a gold-coated single pin, multiple pins, and solid-gel electrodes. The results confirmed the high quality achieved by dry electrodes. They offered the same levels as wet electrodes with significantly reduced times of montage and increased the comfort of users. Although the signal quality is inevitably reduced, the dry electrodes are a reliable system for non-clinical and goal-oriented investigations, such as a comparison between two different mental states during real flight conditions.

#### EEG in Aeronautics

Since the late 1950s, many studies have investigated changes in EEG rhythms during flight conditions (Carl et al., 1959) despite the noisy environment (e.g., vibration, wind, acoustic noise, physiological artifacts, and important pilot physical movements). The recorded EEG signals show specific features and changes in the power spectrum of the various frequency bands associated with flight performance (Carl et al., 1959). One EEG experiment (Callan et al., 2015) conducted under real flight conditions, showed that the neural signature of inattentional deafness (e.g., inadvertently missing an auditory stimulus) was revealed by a reduction in phase resetting in the alpha and theta band frequencies. Sauvet et al. (2014) have used a single EEG channel during real long-haul flights to detect low states of vigilance. Another EEG study involved a critical scenario in a flight simulator (Dehais et al., 2016), which detected the existence of an early and unconscious gating mechanism based on the association between the auditory alarm and the N100 and P300 amplitude. Di Stasi et al. (2016) analyzed the in-flight EEG activity of military helicopter pilots during real flights. They found that highly demanding procedures related to takeoff and landing were associated with higher power EEG frequency bands, whereas less demanding procedures were associated with lower EEG power over the same frequency bands. Several EEG investigations have focused on the mental workload of the operator during a flight task execution. For example, EEG revealed variations in alertness and could predict lower performance caused by increased mental workload during flight operations (Borghini et al., 2014). Theta changes were observed over the frontal brain areas when comparing the training improvements of novice pilots in flight simulation tasks (Borghini et al., 2014).

Workload perception was shown to be dependent on the level of experience, the abilities, or just the individual differences between pilots. The EEG was sensitive to workload level variance between novice and expert aircraft pilots during the execution of the identical task (Doppelmayr et al., 2008; Parasuraman and Jiang, 2012).

Recent studies exploring Air Traffic Controllers (Bernhardt et al., 2019) have computed an EEG-based workload index that could differentiate between task workload requirements exploring front-parietal brain function. Yet, EEG exploration has achieved traction in aviation and space operations, current studies face challenges related to the intrusive and bulky nature of the equipment (Caldwell et al., 2002), the discomfort of long preparation time, and dependence on gel electrodes (e.g., wet electrodes). In a recent study, Dehais et al. (2019) tested a dry EEG system on pilots during the low and high load traffic patterns associated with the passive auditory oddball. Analysis of ERPs and frequency characteristics has confirmed that dry EEG can be used to study cognition during highly ecological and noisy conditions. Recent developments in dry electrodes (Liao et al., 2012) allow them to reduce preparation time by eliminating the conductive gel or saline patch and preparing the skin to reduce the contact impedance. Some dry electrodes use a ring model with pins to touch the scalp (Hairston et al., 2014), while other dry electrodes use foam-based supplies covered in conductive textile materials. Other promising non-contact electrode techniques enable weak biopotentials to be amplified using a contact-free electrode-skin.

### Electro-Oculograghy (EOG)

#### Origin of EOG Response

**Figure 1:**
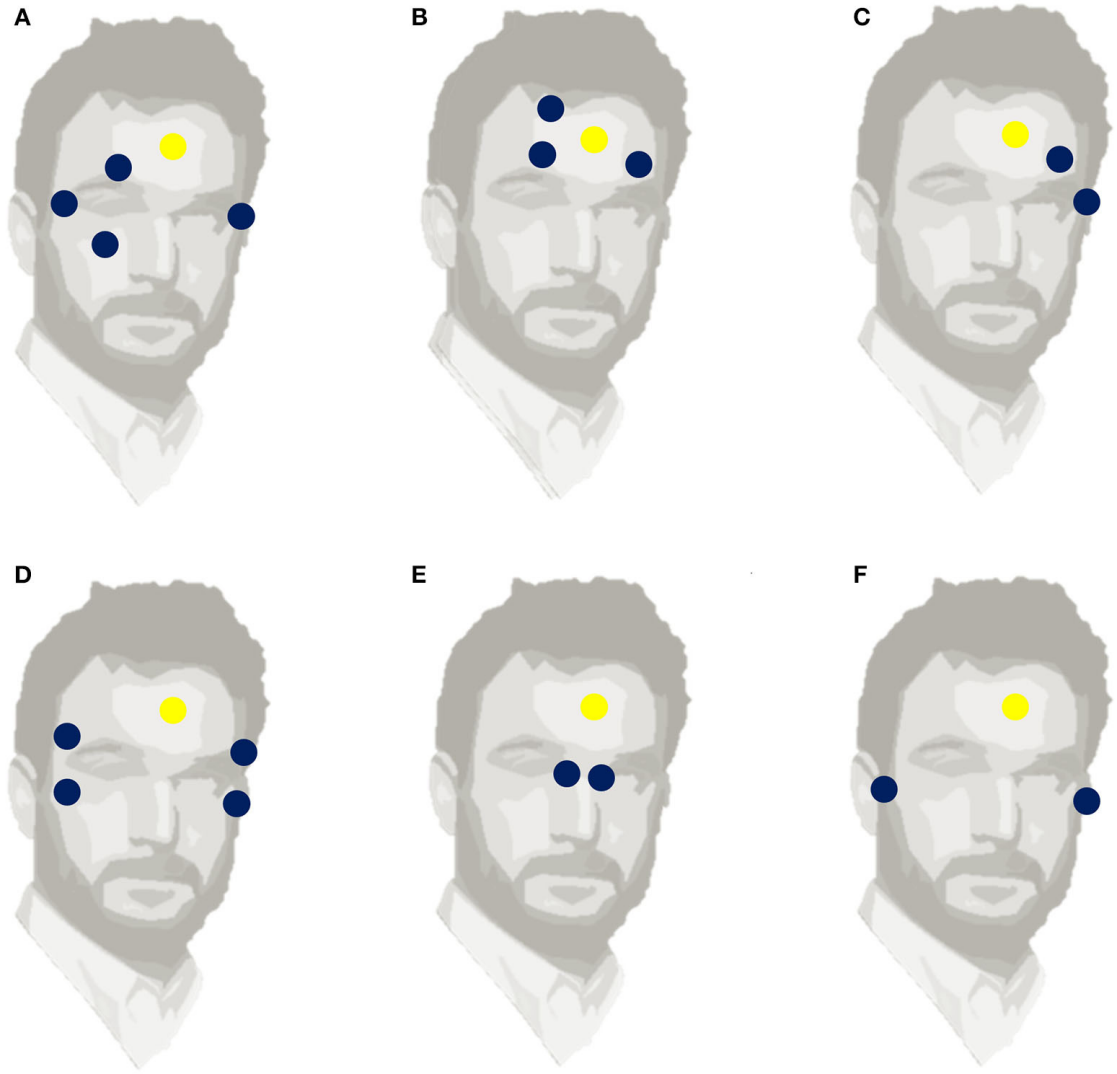
Six different electrode positions, used to measure EOG. The blue circles indicate the locations of horizontal and vertical EOG electrodes. The yellow circle **(A)** Standard placement with 4 electrodes, **(B)** Frontal placement, **(C)** One-side two-electrode placement, **(D)** Two-side symmetrical 4-electrode placement, **(E)** Nasal two-electrode placement, **(F)** Near-ear two-electrode placement indicates the reference electrode.

The EOG method recognizes the differences in potential changes induced by eye movements between two electrodes placed either horizontally or vertically around the eyes. Early eye movement studies revealed that the human eye is an electric dipole, comprising a positively charged cornea and a negatively charged retina (Anderson, 1937). When the eyeball moves in the direction of the electrode, the electrical potential increases and it decreases when the eye moves in the other direction. The voltage variation between the poles is known as corneoretinal potential and varies between 0.4 and 1 mV. The acquired potential varies based on the viewing angle, up to an angle of 30 degrees (Anderson, 1937). When the eyes do not move and are fixed, the potential does not vary. When the eye moves in the direction of the sensor, the potential greatly increases. Importantly, for people who are blind, or when people have their eyes are closed, the electrical changes remain. There are many configurations for electrode placement. Commonly, the horizontal electrodes are placed at the external borders of the eyes while the vertical ones are positioned above and below the eye (Singh and Singh, 2012). The horizontal EOG signal is the difference of the voltage between horizontal electrodes and the vertical EOG signal is the subtraction of the voltage between the vertical electrodes. Usually, the reference electrode is placed on the forehead (Barea et al., 2002) in the middle of the eyes (Yamagishi et al., 2006) or on the mastoids (Pettersson et al., 2013). Other atypical electrode placements have been extensively tested such as six electrodes positioned above and below both the eyes (Pettersson et al., 2013). Some typical and atypical electrode placements are presented in Figure 1.

Other studies have used a headband to place electrodes close to the skin and minimize noise, but without measuring the vertical eye movements (Chang, 2019). Adopting this technique, Yan et al. (2013) positioned five electrodes, one horizontal and one vertical around each eye in addition to the reference. Kanoh et al. (2015) placed electrodes near nasion and on both sides of the rhinion, which correspond to the bridge and nose pads of eyewear. Another remarkable placement was proposed by Manabe et al. (2013), who pioneered in-ear EOG signal detection by testing different material- electrode combinations that are suitable for daily use. They highlighted the relation between the in-ear electrical signal and eye movement but did not estimate the accuracy of the eye position. Favre-Felix et al. (2017) proposes a novel fitted ear EOG device that uses a single model taken from the participant's ear canals. Interestingly, they found a strong correlation between conventional EOG and ear EOG signals. Hence, ear EOG can be used to accurately estimate eye gaze in real-time situations, which is particularly relevant for aeronautics and the hearing aid industry. For example, a visually directed hearing aid could be faster and easier to manipulate than a hearing aid piloted by other conventional tools like a pointer or remote control (Hart et al., 2009).

In addition to these techniques, numerous tests have explored the placement options for mobile electrodes that measure changes in EOG voltage. JinsMeme (JINS Inc., Japan) implanted an EOG amplifier into eyeglasses. BioPac (CA, USA) connected a mobile EOG device to a stationary system (MP160) with wireless communication. Some other EOG systems have been connected to a wheelchair (Rajesh, 2014) or fixed into goggles that can be used in everyday environments (Bulling et al., 2009). One study (Acuna et al., 2014) has shown that a low-cost EOG system (<50 euros) can give good results for eye tracking. The performance achieved by this system is different depending on whether one considers vertical or horizontal movements; the monitoring of the latter being much more precise. Thus, an EOG system gives an accuracy of <3° on the horizontal plane (with movements ranging from −50° to +50°), and an accuracy of <4° on the vertical plane (movements ranging from −10° to +10°). For larger vertical movements (from −30° to +30°), the imprecision increases to 11°. Other systems could be used as an alternative way of measuring combined EEG and EOG including Muse (Toronto, Canada), Melomind (Paris, France), Open BCI (Brooklyn, NY, USA), and Emotiv system (San Francisco, USA). They present several advantages over conventional wired, bulky EEG devices in that they are economical, portable, and easy to use. In summary, the main reason for such diversity in electrode placement is due to the specificity of each experimental paradigm and whether other equipment is associated with EOG. Largely, these systems recognize eye movements with accuracy and further investigation of electrode placement and the quality of the EOG signal will be the subject of future research.

#### EOG Approaches

The main eye movements captured with EOG are fixations and saccades (Singh and Singh, 2012). Fixational movement is an attempt to capture the steady image on the retina. Depending on the quality of the processed information and the current cognitive load, fixation can last between 100 and 1,000 ms, with a mean range of 200–500 ms. The saccade movement changes the eye direction around the field of view and brings the object of interest into the foveal region. Saccades are characterized by a simultaneous and rapid change of the eye position between two fixation points. The duration of saccades is determined by the angular distance the eye travels during the movement. The saccades involve distinct patterns in the EOG signal. They are relatively easy to identify as the deflected amplitudes are above the common high-frequency noise level, and they are short (in duration). When the target is slowly moving, smooth pursuit movement allows us to maintain focus. Smooth pursuit eye movements are typically initiated with a delay of 100–180 ms relative to the onset of an unpredictable motion (Lisberger and Westbrook, 1985). It is difficult to distinguish these movements on EOG recordings because it can be confounded with linear signal drift. Other movements (such as vestibulo-ocular movements and optokinetic nystagmus) allow for the gaze to stabilize during head and body movement. Nystagmus is a rhythmic, often rapid, involuntary eye movement that takes place when the head moves rapidly and the eyes move in response to the moving fluid in the vestibular system (Kang and Shaikh, 2017). Physiological nystagmus occurs during the motions of the head (vestibular nystagmus), or in the presence of patterns in the visual field (optokinetic nystagmus). It is often accompanied by a feeling of disorientation or vertigo and can be a reliable indicator of vestibular pathology. Vergence movements rotate the eyes inwards and outwards, with slow 10°/s disconjugate movements. They allow the visual system to incorporate deep targets, permitting the perception of the world across three dimensions (Alvarez et al., 2005). Even if the eyeballs participate in the contraction of medial and lateral rectal muscles until the paired images are projected onto the fovea, they will activate shared and independent nerve regions. While saccadic and vergence eye movements rely on different muscles to restore the globes, they will activate both shared and distinct neural areas (Semmlow et al., 1998).

Numerous algorithms exist for detecting and modeling oculomotor parameters including eye blink, saccade direction, and fixation. Nolan et al. (2010) developed a fully automated statistical thresholding method that detects and classifies a portion of the signal as an eye blink if the potential of the portion exceeds the threshold. Niemenlehto (2009) analyzed EOG signals by exploring a detection method of a constant false alarm rate that detects saccades. Pettersson et al. (2013) detected eye blinks and saccades by identifying the threshold of the temporal derivative of the EOG signal. Behrens et al. (2010) explored the deviation of the eye-movement acceleration of the EOG signals. Toivanen et al. (2015) computed a real-time algorithm EOG signal that automatically detects blinks, saccades, and fixations and analyzes the temporal features of these reflexes. Although EOG signals have a good signal-to-noise ratio, due to their large amplitude and the relative ease with which they can detect saccades and blinks (Skotte et al., 2007), they are continuously contaminated by physiological artifacts such as electromyography (EMG). EMG artifacts are detected when the participant moves their facial muscles or body during EOG recording, such as jaw clenching, raising an eyebrow, or smiling. The forms and amplitudes of these artifacts depend on the types of movement, the position of the electrode, and sampling rates. High-frequency noise and smooth waveforms are beyond the potential range of eye movement-related signals and are usually differentiated. Median and low-pass filters are the most commonly used techniques to preserve these noises while retaining edge. The filters are based on split windows with varied cut-off frequencies and can be used for either online and/or offline analysis.

Besides EOG, other eye-tracking techniques include photo- and video-oculography, or scleral research lenses (Duchowski, 2017). These techniques allow the capture of eye movements with greater precision than EOG but involve complex set-ups (flight deck video-camera integration, for instance) and involve processing pipelines that demand a large amount of power (computer vision algorithms). Alternatively, EOG is a technique associated with simple electrodes that can be embedded in a headset, providing good insights into the mental state and brain activity of a participant with lower consumption of energy and simpler processing pipelines, which are useful in aeronautics.

#### EOG in Aeronautics

The assessment of eye movements is particularly relevant for aeronautic (Peysakhovich et al., 2018) and neuro-ergonomic applications (Peysakhovich et al., 2019). Given the EOG's capacity to detect eye movements, it is an excellent candidate to be embedded into aeronautical systems. A number of important studies have related eye movement analysis to fatigue, mental workload, and cognitive performance in pilots. One of the most important causes of aviation accidents is provoked by human errors e.g., drowsiness or fatigue (Velazquez, 2018). Pilot fatigue and loss of control are considered by the Federal Aviation Administration as one of four common aviation hazards (Federal Aviation Administration, “Risk Management Handbook: U.S. Department of Transportation,” 2016). For example, a study described how two pilots from a commercial aircraft missed the target airport because both were sleeping (Borghini et al., 2014). Thus, EOG is considered to be an effective and predictive tool in detecting drowsiness markers such as a reduction in performance, and changes in the frequency of eye blinking (Oken et al., 2006). Interestingly, eye movement analysis can serve as a reference for the mental workload and state of pilots (Di Nocera et al., 2007). It has been shown that the workload reflected by eye fixation distribution varies according to the phases of the flight. The highest workload was noted during takeoff and landing, and the lowest during the cruise phase (Di Nocera et al., 2007). Brams et al. (2018) discuss how the gaze behavior of expert pilots differs from that of novices, suggesting relevant information about basic processes that explain the successful performance of expert pilots in flight. The authors explain that the expert pilots have expanded visual range that analyzes the global scene using the field of view next to the fovea, and that they then shift their attention to the pertinent area (Gegenfurtner et al., 2011). Experts have larger saccades that cover more areas and spend less time focusing on task-related regions. In addition to the important capacity to process information from multiple sources, this ability is also related to advanced cognitive performances. The gaze behavior of fighter pilots during flight conditions also varies according to altitude and speed (Svensson et al., 1997). An augmented workload can induce a lower percentage of eye fixation distribution outside and a higher percentage of fixation on the tactical display.

In addition to investigating the eye movement patterns underlying cognitive functions, alternative EOG (around the ear) may be a promising tool in real aeronautic environments. In numerous activities such as air traffic control or piloting an aircraft, the operators are equipped with peripherals (typically headsets). As the EOG requires only a few electrodes, it does not obstruct the visual field nor does it unnecessarily illuminate the eyes with infrared light. Therefore, this technique is convenient for head-mounted peripherals such as the audio headset used by pilots. Moreover, exploring EOG integration in control and communication peripherals may enhance human-system interaction and make psycho-physiological monitoring possible (e.g., based on blink rate or saccades). Such an approach could have numerous applications in aeronautics (fighters, helicopters, and unmanned aerial vehicle operation), naval systems, and control-command centers.

### Motivations and Contributions

Although a large number of surveys are published for EEG and EOG in several fields, to the best of our knowledge, we have not identified any exhaustive review that summarizes a taxonomy of combined EEG-EOG studies. To bridge this gap, this work analyzes and categorizes developments from the last decade, examining published literature on combined EEG-EOG in different applications related to aeronautics.

This non-exhaustive review analyzes literature from the oldest to the most recent relevant studies. Section Introduction presents EEG and EOG signals, their methodological approaches, and a summary of their application in aeronautics. This section could be particularly beneficial for beginners in the field of electrophysiology.

The review presents a novel taxonomy model for classifying different approaches to EEG and EOG by emphasizing the extracted features, the applied pre-processing treatment, and the performance metrics. The studies belonging to each main category are sub-categorized as per the corresponding domain of application. Sections Methodology of the Review and Results are particularly beneficial for expert researchers in the field. Sections Discussion and Limitations and Conclusions Outline Insights and research directions as a means of providing guidelines for beginner and expert researchers who are interested in combined EEG-EOG studies in the future.

This review is useful for both beginners and experts in this field. It is intended to be a time- and resource- saving guide for those searching for exploration of neuro-physiological correlates in aeronautics. On the one hand, this review is beneficial for beginners who may easily explore specific literature on EEG and EOG from basic approaches to aeronautics applications in a single document and investigate it by spending less effort. On the other hand, it is useful for expert researchers who may explore the literature to discover trends and methodologies for exploring brain and eye signals. Experts may explore these approaches as valuable tools and could be useful in building and analyzing experimental paradigms.

## Methodology of the Review

Peer-reviewed journal articles in the English language and conference papers published on PubMed for the decade from 2010 to 2020 (January 2010-March 2020) were identified as targets for our review. The database was last queried on March 23, 2020. Search items related only to studies combining EEG and EOG methods in experiments. This filtered selection resulted in 249 studies that were then included or excluded according to subsequent criteria. The inclusion criteria were: (i) combined EEG-EOG studies; (ii) original research papers; and, (iii) that the experiments involved human subjects. The exclusion criteria were: (i) review, methodology, and proof-of-concept papers; (ii) dataset publications; (iii) animal model experiments; and, (iv) studies focusing solely on EEG or EOG.

Article titles were examined to evaluate the relevance of a selected article. If the title did not noticeably specify whether it corresponds to the inclusion and exclusion criteria, the abstract was then considered. Lastly, during the full paper examination and data gathering process, an article that did not meet the criteria was excluded. Non-peer reviewed papers, such as arXiv or BiorXiv electronic preprints were considered as a possible source of bias. Thus, preprints that had not been peer-reviewed were not selected. Finally, 150 studies were included in our final database. We constructed a data extraction table containing several data items related to our investigation question, according to previous comments with a similar scope and the author's past work in the field.

A taxonomy table was used to classify and describe the included items for each selected study. The first section is the justification category that emphasizes the domains of application of the included studies (e.g., sleep, BCI, signal processing, cognition, and driving). This valuable information allows an understanding of the scope of the research and also enabled us to determine trends in the analysis. The second section shows the bibliographic reference attributed to each study. The third section shows the name of the first author, giving a specific identification for each paper in the database. The fourth section outlines the year of publication, which is relevant for our selection criteria and statistical analysis. The fifth section regroups the number of subjects in each study to give a quick overview of the coherence of the relative main findings. The sixth section covers the publication category of the article, such as whether it was a journal article or a conference publication. These first six sections indicate the types of included papers and the main selected items. The seventh section includes all relevant information about the EEG and EOG data. This comprises the category of EEG and EOG equipment used, in addition to the different extracted features.

Standard EEG and standard EOG sections refer to classic wet electrode gels that are not mobile, while non-standard refers to dry wearable electrodes and any other equipment that is different from the standard category. Features Classification (Table 1) refers to the properties of the analyzed EEG and EOG signal. The content of this dataset includes the support of the statistical analysis relevant to the critical component of our discussion. The EEG features an extraction section and covers the signal processing methodology that was analyzed in each study, including the waveband frequency Delta (2–4 Hz), Theta (4–8 Hz), Alpha (9–13 Hz), Beta (14–32 Hz), Gamma (over 33 Hz), the ERP, or the basic raw signal. The EOG feature extractions consist of the explored signal processing for eye movements including blinks, saccades, fixations, raw signal, or whether the EOG was used as an artifact handling methodology. Polysomnography is a multi-parameter test based on several different types of physiological signals called polysomnograms used in sleep diagnosis. Here, polysomnography was included in the taxonomy as an extracted feature from sleeping studies that analyzed both EEG and EOG signals.

**Table 1:** Taxonomy table.

						EEG		EEG features								EOG		EOG features						Pre-proc.				Perf. metrics					

Domain	Author	Year	# subjects	Journal	Conference	Standard	Non standard	Alpha	Beta	Theta	Gamma	Delta	ERPs	Raw signal	Polysomn.	Standard	Non standard	Blinks	Saccades	Fixations	Raw signal	Art. removal	Polysomn.	Filtring	Art. handling	Downsampling	Not mentioned	Sens./Spec.	Class. score	Error corr.	FP/FN	Accuracy	Other	Reference
	Lee	2018	20	•	°	•	°	°	°	°	°	°	•	°	°	•	°	•	°	°	°	°	°	•	°	•	°	°	•	•	°	•	°	Lee et al. (2018)
	He	2019	10	•	°	•	°	°	°	°	°	°	•	°	°	•	°	•	°	°	°	°	°	•	°	°	°	°	•	°	•	•	°	He et al. (2017)
	Zhang	2019	6	•	°	•	°	°	°	°	°	°	°	°	°	•	°	°	°	°	°	°	°	•	°	°	°	°	°	°	°	•	°	Zhang et al. (2019b)
	Soekada	2015	6	•	°	°	•	°	°	°	°	°	°	•	°	•	°	°	°	°	•	°	°	•	°	°	°	°	°	°	°	°	•	Soekadar et al. (2015)
	Huang	2019	10	•	°	°	•	°	°	°	°	°	°	•	°	°	•	•	°	°	°	°	°	•	°	°	°	°	°	°	•	•	°	Huang et al. (2019)
	Punsawad	2010	3	°	•	•	°	•	•	°	°	°	°	°	°	•	°	°	°	°	•	°	°	°	°	°	•	°	°	°	°	•	°	Punsawad et al. (2010)
	Usakli	2010	10	•	°	•	°	°	°	°	°	°	•	°	°	•	°	•	°	°	•	°	°	•	°	°	°	°	°	°	°	°	•	Usakli et al. (2010)
	Hsu	2011	5	•	°	•	°	°	°	°	°	°	•	°	°	•	°	°	°	°	°	•	°	•	•	°	•	•	°	°	•	•	°	Hsu (2011)
	Breitwieser	2011	NM	°	•	•	°	°	°	°	°	°	°	•	°	•	°	°	°	°	•	°	°	°	°	°	•	°	°	°	°	°	•	Breitwieser et al. (2011)
	Liu	2011	8	•	°	•	°	°	°	°	°	°	•	°	°	•	°	•	°	°	°	°	°	•	°	°	°	°	°	•	°	•	°	Liu et al. (2011)
	Wang	2012	9	•	°	•	°	°	°	°	°	°	°	•	°	•	°	°	°	°	°	•	°	•	•	•	°	°	°	°	°	•	°	Wang et al. (2012)
	Hsu	2013	8	•	°	•	°	°	°	°	°	°	°	•	°	•	°	°	°	°	•	•	°	•	•	•	°	°	°	°	°	•	°	Hsu (2013b)
	Aziz	2014	20	•	°	°	•	•	°	°	°	°	°	•	°	°	•	•	°	°	°	°	°	•	°	•	°	°	°	•	°	•	°	Aziz et al. (2014)
BCI	Jiang	2014	4	•	°	•	°	°	°	°	°	°	°	•	°	°	•	°	°	°	•	°	°	•	°	•	°	°	°	°	°	•	°	Jiang et al. (2014)
	Wang	2014	4	•	°	°	•	°	°	°	°	°	•	°	°	•	°	•	°	°	°	°	°	•	°	°	°	°	°	°	•	•	°	Wang et al. (2014a)
	Witkowski	2014	12	•	°	•	°	°	°	°	°	°	°	•	°	•	°	°	°	°	•	°	°	•	°	•	°	•	°	°	•	•	°	Witkowski et al. (2014)
	Ma	2015	13	•	°	•	°	°	°	°	°	°	•	°	°	•	°	•	°	°	°	°	°	•	°	•	°	°	°	•	°	•	°	Ma et al. (2015)
	Daly	2015	13	•	°	•	°	•	°	°	•	°	°	•	°	•	°	•	°	°	°	°	°	°	•	•	°	•	°	°	•	•	°	Daly et al. (2015)
	Min	2016	20	•	°	•	°	°	°	°	°	°	°	•	°	•	°	°	°	°	•	°	°	°	°	•	°	°	°	°	°	•	°	Min et al. (2016)
	Kumar	2016	10	•	°	•	°	°	°	°	°	°	°	•	°	•	°	•	•	•	°	°	°	°	°	°	•	°	•	°	°	°	•	Kumar et al. (2016)
	He	2017	5	°	•	°	°	°	°	°	°	°	•	°	°	°	°	•	°	°	°	°	°	•	°	°	°	°	•	°	•	•	°	He et al. (2017)
	Li	2017	10	•	°	•	°	•	•	•	•	°	°	°	°	•	°	•	°	°	°	°	°	•	•	°	°	°	°	°	°	•	°	Li et al. (2017)
	Crea	2018	7	•	°	°	•	°	°	°	°	°	°	•	°	°	•	°	°	°	°	•	°	•	°	°	°	•	°	°	•	°	°	Crea et al. (2018)
	He	2018	8	•	°	°	•	•	°	°	°	•	°	°	°	°	•	°	°	°	°	•	°	•	•	°	°	°	°	°	°	°	•	He et al. (2018)
	Ivorra	2018	10	•	°	•	°	°	°	°	°	°	°	•	°	•	°	•	°	°	°	°	°	•	°	°	°	°	•	°	•	•	°	Ivorra et al. (2018)
	Arnin	2018	9	°	•	•	°	°	°	°	°	°	°	•	°	•	°	°	°	°	•	°	°	•	•	°	°	°	°	•	°	•	°	Arnin et al. (2018)
	Yu	2019	18	•	°	•	°	°	°	°	°	°	•	°	°	•	°	•	°	°	°	°	°	•	°	•	°	°	•	°	°	•	°	Yu et al. (2019)
	Badesa	2019	11	•	°	°	•	°	°	°	°	°	•	°	°	°	•	°	°	°	•	°	°	•	°	°	°	°	°	°	°	°	•	Badesa et al. (2019)
	Al-Hudhud	2019	2	•	°	•	°	•	•	•	°	°	°	°	°	•	°	•	°	°	°	•	°	•	•	°	°	°	•	°	°	°	°	Al-Hudhud et al. (2019)
	Han	2020	12	•	°	°	•	°	°	°	°	°	°	•	°	°	•	•	°	°	•	°	°	•	•	°	°	°	°	°	•	•	°	Han et al. (2020)
	Zhou	2020	10	•	°	•	°	°	°	°	°	°	°	•	°	•	°	•	°	°	°	°	°	•	°	°	°	°	°	°	•	•	°	Zhou et al. (2020)
	Javaid	2010	15	•	°	•	°	°	°	°	°	°	°	°	°	•	°	°	•	•	°	°	°	•	•	°	°	•	°	°	°	°	•	Javaid et al. (2010)
	Killane	2013	7	°	•	•	°	°	°	°	°	°	•	°	°	•	°	°	°	°	°	•	°	•	°	°	°	°	°	°	°	°	•	Killane et al. (2013)
	WU	2013	16	•	°	•	°	•	•	•	•	•	°	°	°	•	°	°	°	°	•	°	°	•	•	°	°	°	°	°	°	°	•	Wu et al. (2013)
	Tang	2013	46	•	°	•	°	°	°	°	°	°	•	°	°	•	°	•	°	°	°	•	°	•	•	°	°	•	°	°	°	•	°	Tang et al. (2013)
	Kaur	2013	90	•	°	•	°	°	°	°	°	°	°	•	°	•	°	°	°	°	°	•	°	•	°	°	°	°	°	°	°	°	•	Kaur et al. (2013)
	Justen	2014	14	•	°	•	°	•	•	•	°	•	°	°	°	•	°	°	°	°	•	°	°	•	•	°	°	°	°	°	°	°	•	Justen et al. (2014)
Cognition	Keshavarz	2014	13	•	°	•	°	°	°	°	°	°	•	°	°	•	°	°	°	°	•	°	°	•	°	°	°	°	°	°	°	°	•	Keshavarz and Berti (2014)
	Zheng	2016	23	•	°	•	°	•	•	•	•	°	°	°	°	•	°	•	•	•	°	°	°	•	°	•	°	°	°	•	°	°	•	Zheng and Lu (2016)
	Hohyun Cho	2016	32	•	°	•	°	°	°	°	°	°	•	°	°	•	°	•	°	°	°	°	°	•	°	°	°	°	°	°	°	°	•	Cho et al. (2016)
	Borghini	2016	10	•	°	•	°	•	°	•	°	°	°	°	°	•	°	•	°	°	°	°	°	•	•	°	°	°	°	°	°	•	°	Borghini et al. (2017)
	Meyberg	2017	32	•	°	•	°	°	°	°	°	°	•	°	°	•	°	•	•	°	°	°	°	•	•	°	°	°	°	°	°	°	•	Meyberg et al. (2017)
	Chang	2016	24	•	°	•	°	•	°	°	°	°	°	°	°	•	°	•	°	°	°	°	°	•	•	°	°	°	•	°	•	•	°	Chang et al. (2016)
	Hsu	2013	6	•	°	•	°	°	°	°	°	°	•	°	°	•	°	°	°	°	°	•	°	•	•	°	°	°	°	°	°	•	°	Hsu (2013a)
	Chan	2010	8	•	°	•	°	°	°	°	°	°	°	•	°	•	°	°	°	°	•	°	°	•	•	°	°	°	•	°	°	°	°	Chan et al. (2010)
	Gao	2010	17	•	°	•	°	°	°	°	°	°	°	•	°	•	°	°	°	°	•	°	°	•	•	°	°	•	°	°	°	•	°	Gao et al. (2010)
	Pham	2010	24	•	°	•	°	°	°	°	°	°	°	°	°	•	°	•	°	°	•	°	°	•	•	°	°	°	°	°	°	°	•	Pham et al. (2011a)
	Sommer	2010	16	°	•	•	°	°	°	°	°	°	°	•	°	•	°	°	°	°	•	°	°	°	°	°	•	°	°	•	°	•	°	Sommer and Golz (2010)
	Zhang	2010	1	°	•	°	•	•	°	°	°	°	°	°	°	°	•	°	°	°	•	°	°	°	°	°	•	°	°	°	°	°	•	Zhang et al. (2010a)
	Ma	2010	5	°	•	•	°	°	°	°	°	°	°	•	°	•	°	•	°	°	°	°	°	•	°	•	°	°	°	•	°	°	•	Ma et al. (2010)
	Zhang	2010	4	•	°	•	°	•	•	•	•	•	°	°	°	•	°	°	°	°	°	•	°	•	•	°	°	°	°	°	°	•	°	Zhang et al. (2010b)
	Pham	2011	24	•	°	•	°	°	°	°	°	°	•	°	°	•	°	•	°	°	•	°	°	•	•	°	°	°	°	°	°	°	•	Pham et al. (2011b)
	Ma	2011	28	•	°	•	°	°	°	°	°	°	°	•	°	•	°	°	°	°	•	°	°	•	•	°	°	°	°	•	°	°	°	Ma et al. (2011)
	Babiloni	2011	4	•	°	°	•	•	°	°	°	°	°	•	°	•	°	•	°	°	°	°	°	°	•	°	°	°	°	°	°	°	•	Babiloni et al. (2011)
	Khalighi	2011	14	°	•	•	°	°	°	°	°	°	°	°	•	•	°	°	°	°	°	°	•	•	•	°	°	•	°	°	•	•	°	Khalighi et al. (2011)
	Kirenskaya	2011	42	•	°	•	°	°	°	°	°	°	°	•	°	•	°	°	•	°	•	°	°	•	•	°	°	°	°	•	°	°	°	Kirenskaya et al. (2011)
Signal processing	Noureddin	2012	4	°	•	•	°	°	°	°	°	°	°	•	°	•	°	•	°	•	°	•	°	°	•	°	°	°	°	°	°	°	•	Noureddin et al. (2012)
	Noureddin	2012	13	•	°	•	°	°	°	°	•	°	°	•	°	•	°	•	•	°	°	•	°	•	•	°	°	°	°	°	•	°	°	Carl et al. (2012)
	Carl	2012	3	•	°	•	°	•	•	•	•	•	°	°	°	•	°	°	°	°	°	•	°	•	•	°	°	°	°	•	°	•	°	Cannon et al. (2012)
	Cannon	2012	NM	°	•	•	°	°	°	°	°	°	°	•	°	•	°	°	°	°	•	°	°	•	°	°	°	°	°	•	°	°	°	Casson and Rodriguez-Villegas (2012)
	Casson	2012	15	•	°	•	°	°	°	°	°	°	°	•	°	•	°	•	°	°	•	°	°	•	•	°	°	°	°	°	°	•	°	Kong et al. (2013)
	Kong	2013	NM	°	•	•	°	°	°	°	°	°	°	•	°	•	°	•	°	°	°	°	°	•	°	°	°	°	°	•	•	°	°	Bizopoulos et al. (2013)
	Tan	2013	12	°	•	•	°	°	°	°	°	°	°	•	°	•	°	°	°	°	•	°	°	•	•	°	°	•	°	°	°	•	°	Tan et al. (2013)
	Kim	2013	144	•	°	•	°	•	•	•	•	•	°	°	°	•	°	•	°	°	°	°	°	•	•	•	°	°	°	°	°	°	•	Kim et al. (2013)
	Klein	2013	55	•	°	•	°	°	°	°	°	°	•	°	°	•	°	•	°	°	•	•	°	•	°	°	°	°	°	°	•	°	°	Klein and Skrandies (2013)
	Pettersson	2013	3	•	°	•	°	°	°	°	°	°	°	•	°	•	°	•	•	°	°	°	°	•	•	°	°	•	°	•	°	°	•	Pettersson et al. (2013)
	Zeng	2014	40	•	°	•	°	°	°	°	°	°	°	•	°	•	°	°	°	°	•	°	°	•	•	°	°	°	°	°	°	°	•	Zeng and Song (2014)
	Barry	2014	20	•	°	•	°	•	•	•	•	°	°	°	°	•	°	°	°	°	°	•	°	•	°	•	°	°	°	°	°	°	•	Barry et al. (2015)
	Sameni	2014	NM	•	°	•	°	°	°	°	°	°	°	•	°	•	°	°	°	°	•	°	°	•	•	°	°	°	°	°	°	°	•	Sameni and Gouy-Pailler (2014)
	Jaleel	2014	2	•	°	•	°	°	°	°	°	°	°	•	°	•	°	°	°	°	°	•	°	•	°	°	°	•	•	•	•	•	°	Jaleel et al. (2014)
	Bou assi	2014	3	°	•	•	°	°	°	°	°	°	°	•	°	•	°	°	°	°	°	•	°	•	•	°	°	°	°	°	•	•	°	Assi et al. (2014)
	Torres-Valencia	2014	32	°	•	•	°	•	•	•	•	°	°	°	°	•	°	•	°	°	°	°	°	°	°	°	•	°	°	•	°	°	°	Torres-Valencia et al. (2014)
	Verma	2014	32	•	°	•	°	°	°	°	°	°	°	•	°	•	°	°	°	°	•	°	°	°	°	°	°	°	•	°	°	•	°	Verma and Tiwary (2014)
	MacDonald	2014	16	•	°	•	°	°	°	°	°	°	•	°	°	•	°	°	°	°	°	•	°	°	°	°	•	°	°	°	•	°	°	MacDonald and Barry (2014)
	Lee	2014	75	•	°	•	°	°	°	°	°	°	•	°	°	•	°	°	°	°	°	•	°	•	•	°	°	°	°	°	°	°	•	Lee et al. (2014)
	Laszlo	2014	6	•	°	•	°	°	°	°	°	°	•	°	°	•	°	°	°	°	°	•	°	•	°	°	°	°	°	°	°	°	•	Laszlo et al. (2014)
	Hsu	2015	9	•	°	•	°	°	°	°	°	°	°	•	°	•	°	°	°	°	°	•	°	•	•	°	°	°	°	°	°	•	°	Hsu (2015)
	Winkler	2015	21	°	•	•	°	°	°	°	°	°	°	°	°	•	°	°	°	°	°	•	°	•	•	•	°	°	°	°	°	•	°	Winkler et al. (2015)
	Gordon	2015	10	•	°	•	°	•	•	°	°	°	•	°	°	•	°	•	°	°	°	•	°	•	•	°	°	°	°	°	°	°	•	Gordon et al. (2015)
Signal processing	Chang	2015	24	•	°	•	°	°	°	°	°	°	°	•	°	•	°	•	°	°	°	•	°	•	•	•	°	°	°	•	•	•	°	Chang et al. (2016)
	Wang	2016	3	•	°	•	°	°	°	°	°	°	°	•	°	•	°	•	°	°	°	•	°	•	•	°	°	°	°	•	°	°	°	Wang et al. (2016)
	Di Flumeri	2016	10	°	•	•	°	°	°	°	°	°	°	•	°	•	°	°	°	°	°	•	°	•	°	°	°	°	°	°	°	°	•	Di Flumeri et al. (2016)
	Bai	2016	10	•	°	•	°	°	°	°	°	°	•	•	°	•	°	•	°	°	°	•	°	•	•	°	°	°	°	•	°	°	•	Bai et al. (2016)
	Javed	2017	11	•	°	°	•	°	°	°	°	°	•	°	°	°	•	°	°	°	°	•	°	•	°	°	°	°	°	°	°	°	°	Javed et al. (2017)
	Liu	2017	5	•	°	°	•	°	°	°	°	°	°	•	°	°	•	°	°	°	°	•	°	•	•	°	°	°	°	°	°	•	°	Liu et al. (2017)
	Kleifges	2017	40	•	°	•	°	°	°	°	°	°	°	•	°	•	°	•	•	°	°	°	°	•	•	°	°	°	°	°	•	•	°	Kleifges et al. (2017)
	Barthélemy	2017	15	•	°	•	°	°	°	°	°	°	•	°	°	•	°	•	°	°	°	•	°	•	•	°	°	°	°	°	°	°	•	Barthélemy et al. (2017)
	Delisle-Rodriguez	2017	6	•	°	•	°	°	°	°	°	°	°	•	°	•	°	°	°	°	°	•	°	•	•	•	°	°	°	°	•	•	°	Delisle-Rodriguez et al. (2017)
	Tuncer	2018	NM	•	°	•	°	°	°	°	°	°	°	•	°	•	°	°	°	°	•	°	°	°	°	°	•	°	°	°	°	•	°	Arslan Tuncer and Kaya (2018)
	Zennifa	2018	11	•	°	•	°	•	•	•	•	°	°	°	°	•	°	•	°	°	°	°	°	•	•	°	°	°	°	°	•	•	°	Zennifa et al. (2018)
	Issa	2019	27	•	°	•	°	°	°	°	°	°	•	°	°	•	°	°	°	°	°	•	°	•	•	•	°	•	°	•	•	•	°	Issa and Juhasz (2019)
	Jia	2019	7	•	°	•	°	°	°	°	°	°	°	•	°	•	°	•	•	•	°	°	°	•	°	°	°	°	•	°	•	•	°	Jia and Tyler (2019)
Sleep	Stochholm	2016	18	°	•	°	•	•	•	•	°	°	°	°	°	°	°	°	°	°	°	•	°	•	°	°	°	•	°	°	•	•	°	Stochholm et al. (2016)
	Singh	2014	608	•	°	•	°	°	°	°	°	°	°	°	•	•	°	°	°	°	°	°	•	°	°	°	•	°	°	°	°	°	°	Singh et al. (2014)
	Olsen	2017	853	•	°	•	°	•	°	°	°	°	°	°	•	•	°	°	°	°	°	°	•	•	•	°	°	•	°	°	°	•	°	Olsen et al. (2017)
	Korkalainen	2019	1044	•	°	•	°	°	°	°	°	°	°	°	•	•	°	°	°	°	°	°	•	•	°	•	°	•	°	°	°	•	°	Korkalainen et al. (2019)
	Tagluk	2010	21	•	°	•	°	°	°	°	°	°	°	°	•	•	°	°	°	°	°	°	•	•	•	°	°	°	•	°	°	•	°	Tagluk et al. (2010)
	Haavisto	2010	20	•	°	•	°	°	°	°	°	°	°	•	°	•	°	•	•	°	•	°	°	•	°	°	•	°	°	°	°	°	•	Haavisto et al. (2010)
	Krakovska	2011	20	•	°	•	°	°	°	°	°	°	°	°	•	•	°	°	°	°	°	°	•	•	°	°	°	°	°	•	°	•	°	Krakovská and Mezeiová (2011)
	Kempfner	2011	20	°	•	•	°	°	°	°	°	°	°	°	•	•	°	°	°	°	°	°	•	•	•	°	°	°	°	°	°	°	°	Kempfner et al. (2011)
	Liang	2011	20	°	•	•	°	°	°	°	°	°	°	°	•	•	°	°	°	°	°	°	•	•	°	•	°	•	•	•	°	•	°	Liang et al. (2011)
	Charbonnier	2011	13	•	°	•	°	•	•	•	°	•	°	°	°	•	°	°	°	°	°	°	°	•	•	°	°	°	°	•	°	•	°	Charbonnier et al. (2011)
	Yamaguchi	2011	26	•	°	•	°	•	•	°	°	°	°	°	°	•	°	•	•	°	°	°	°	•	°	°	°	°	°	°	°	°	•	Yamaguchi et al. (2011)
	Christensen	2012	20	°	•	•	°	°	°	°	°	°	°	°	•	•	°	°	°	°	°	°	•	•	°	•	°	•	°	°	°	•	°	Christensen et al. (2012)
	Kempfner	2012	16	°	•	•	°	•	°	°	°	°	°	°	°	•	°	°	°	°	•	°	°	•	•	°	°	•	°	°	•	•	°	Kempfner et al. (2012)
	Pan	2012	20	•	°	•	°	°	°	°	°	°	°	°	•	•	°	°	°	°	°	°	•	•	°	•	°	•	°	°	°	•	°	Pan et al. (2012)
	Khalighi	2012	8	°	•	•	°	°	°	°	°	°	°	°	•	•	°	°	°	°	°	°	•	•	°	°	°	•	°	°	°	•	°	Khalighi et al. (2012)
	Arnin	2013	3	°	•	°	•	°	°	°	°	°	°	•	°	°	•	•	°	°	°	°	°	•	°	°	°	•	°	°	°	•	°	Arnin et al. (2013)
	Kempfner	2013	40	°	•	•	°	•	•	•	•	°	°	°	°	•	°	°	°	°	•	°	°	•	°	°	°	°	°	°	°	•	°	Kempfner et al. (2013)
	Camfferman	2013	41	•	°	•	°	°	°	°	°	°	°	°	•	•	°	°	°	°	°	°	•	°	°	°	•	°	°	°	°	°	•	Camfferman et al. (2013)
	Christensen	2014	115	•	°	•	°	°	°	°	°	°	°	°	•	•	°	°	°	°	°	°	•	•	•	°	°	•	°	°	•	°	°	Christensen et al. (2014)
	Koch	2014	76	•	°	•	°	•	•	•	°	°	°	•	°	•	°	°	°	°	°	•	°	•	°	°	°	°	°	°	°	•	°	Koch et al. (2014)
	Glos	2014	11	•	°	•	°	•	°	°	°	°	°	°	•	•	°	°	°	°	°	°	•	•	•	°	°	°	°	°	°	°	•	Glos et al. (2014)
Sleep	Guenole	2014	10	•	°	•	°	°	°	°	°	°	°	•	°	•	°	°	°	°	°	°	°	•	°	°	°	°	°	°	°	°	°	Guénolé et al. (2014)
	Zhang	2014	20	•	°	•	°	•	•	°	°	°	°	•	•	•	°	°	°	°	°	°	•	•	°	°	°	•	°	°	°	•	°	Zhang et al. (2014)
	Fietze	2015	50	•	°	°	•	°	°	°	°	°	°	•	•	°	•	°	°	°	•	°	•	°	°	°	•	•	°	°	°	•	°	Fietze et al. (2015)
	Kuo	2015	18	•	°	•	°	•	•	°	°	°	°	°	°	•	°	°	°	°	•	°	°	•	•	°	°	°	°	°	°	°	•	Kuo et al. (2016)
	Müller	2015	21	•	°	•	°	°	°	°	°	°	°	°	•	•	°	°	°	°	°	°	•	•	•	°	°	°	°	°	°	°	°	Müller et al. (2015)
	Yaghouby	2015	42	•	°	•	°	°	°	°	°	°	°	°	•	•	°	°	°	°	°	°	•	•	°	°	°	°	•	°	•	•	°	Yaghouby and Sunderam (2015)
	Scarlatelli-Lima	2016	56	•	°	•	°	°	°	°	°	°	°	°	•	•	°	°	°	°	°	°	•	°	°	°	•	°	°	°	°	°	•	Scarlatelli-Lima et al. (2016)
	Rezaei	2017	11	•	°	°	°	•	•	•	°	°	°	°	°	•	°	°	°	°	•	°	°	•	°	°	°	°	°	°	°	°	•	Rezaei et al. (2017)
	Supratak	2017	20	•	°	•	°	°	°	°	°	°	°	•	°	•	°	°	°	°	•	°	°	•	°	•	°	°	•	°	°	•	°	Supratak et al. (2017)
	Reed	2017	9	•	°	•	°	°	°	°	°	°	°	°	•	•	°	°	°	°	°	°	•	•	•	°	°	•	•	°	•	•	°	Reed et al. (2017)
	Klok	2018	100	°	•	•	°	•	•	•	°	•	°	°	•	•	°	°	°	°	•	°	•	•	°	°	°	•	°	°	°	•	°	Klok et al. (2018)
	Shustak	2018	9	•	°	°	•	•	°	°	°	°	°	°	•	°	•	•	°	°	°	°	•	•	•	°	°	°	°	°	°	°	•	Shustak et al. (2018)
	Krauss	2018	40	•	°	•	°	°	°	°	°	°	°	•	°	•	°	°	°	°	°	•	°	•	°	°	°	°	°	°	°	°	•	Krauss et al. (2018)
	Nguyen	2018	18	•	°	•	°	°	°	°	°	°	°	•	°	•	°	°	°	°	•	°	°	•	•	°	°	°	°	°	°	°	•	Nguyen et al. (2018)
	Andreotti	2018	19	°	•	•	°	°	°	°	°	°	°	•	°	•	°	°	°	°	•	°	°	•	°	°	°	•	°	°	°	°	•	Andreotti et al. (2018)
	Whitehead	2018	115	•	°	•	°	•	•	•	°	•	°	°	°	•	°	°	°	°	°	•	°	•	•	°	°	°	°	°	°	°	•	Whitehead et al. (2018)
	Dimitriadis	2018	20	•	°	•	°	•	•	•	•	°	°	•	°	•	°	°	°	°	°	•	°	•	•	°	°	•	•	°	°	•	°	Dimitriadis et al. (2018)
	Zhimin	2018	61	•	°	•	°	°	°	°	°	°	°	°	°	•	°	°	°	°	•	°	°	•	°	°	°	•	°	°	°	•	°	Zhimin et al. (2018)
	Lee	2018	NM	°	•	•	°	°	°	°	°	°	°	°	°	•	°	°	°	°	•	°	°	•	°	°	°	°	°	°	°	•	°	Lee et al. (2018)
	Rosales-Lagarde	2018	13	•	°	•	°	°	°	°	°	°	°	°	•	•	°	°	°	°	°	°	•	•	•	°	°	°	°	°	°	°	•	Rosales-Lagarde et al. (2018)
	Gharbali	2018	10	•	°	•	°	°	°	°	°	°	°	°	•	•	°	°	°	°	°	°	•	•	°	°	°	•	°	°	°	•	°	Gharbali et al. (2018)
	Gunnarsdottir	2018	38	•	°	•	°	°	°	°	°	°	°	°	•	•	°	°	°	°	°	°	•	°	•	°	°	°	°	°	°	•	°	Gunnarsdottir et al. (2018)
Sleep	Koch	2018	27	•	°	•	°	°	°	°	°	°	°	°	•	•	°	°	°	°	°	°	•	•	°	°	°	•	°	°	•	•	°	Koch et al. (2019)
	Guragain	2019	18	°	•	•	°	•	•	•	°	°	°	°	°	•	°	°	°	°	°	•	°	•	•	°	°	•	•	°	°	•	°	Guragain et al. (2019)
	Cooray	2019	106	•	°	•	°	°	°	°	°	°	°	•	°	•	°	°	°	°	•	°	°	•	°	°	°	•	°	°	•	•	°	Cooray et al. (2019)
	Sun	2019	86	•	°	•	°	°	°	°	°	°	°	°	•	•	°	°	°	°	°	°	•	•	°	•	°	°	•	°	°	•	°	Sun et al. (2019)
	Yildirim	2019	61	•	°	•	°	°	°	°	°	°	°	°	•	•	°	°	°	°	°	°	•	•	°	°	°	•	•	°	°	•	°	Yildirim et al. (2019)
	Sokolovsky	2019	20	•	°	•	°	°	°	°	°	°	°	°	°	•	°	°	°	°	°	°	•	•	°	•	°	°	•	°	°	•	°	Sokolovsky et al. (2019)
	Jiang	2019	42	•	°	•	°	°	°	°	°	°	°	°	•	•	°	°	°	°	°	°	°	•	°	°	°	°	•	°	•	•	°	Jiang et al. (2019)
	Gunnarsdottir	2020	22	•	°	•	°	°	°	°	°	°	°	°	•	•	°	°	°	°	°	°	•	•	•	°	°	°	°	°	°	•	°	Gunnarsdottir et al. (2020)
	Khushaba	2011	31	•	°	•	°	°	°	°	°	°	°	•	°	•	°	°	°	°	•	°	°	•	°	°	°	°	•	°	°	•	°	Khushaba et al. (2011)
	Åkerstedt	2013	18	•	°	•	°	•	°	•	°	°	°	°	°	•	°	•	°	°	°	°	°	•	°	°	°	°	•	°	°	°	°	Åkerstedt et al. (2013)
	Hallvig	2014	33	•	°	•	°	•	°	•	°	°	°	•	°	•	°	•	°	°	°	°	°	•	•	°	°	°	•	°	°	°	•	Hallvig et al. (2014)
	Gharagozlou	2015	12	•	°	°	•	•	°	°	°	°	°	°	°	°	•	°	°	°	•	°	°	•	•	°	°	°	•	°	°	°	°	Gharagozlou et al. (2015)
Driving	Nguyen	2016	11	•	°	•	°	•	•	•	•	°	°	°	°	•	°	•	°	°	°	°	°	•	•	•	°	°	°	°	•	•	°	Nguyen et al. (2017)
	Ahn	2016	11	•	°	•	°	•	•	•	•	°	°	°	°	•	°	•	°	°	°	°	°	•	•	°	°	°	°	°	°	•	°	Ahn et al. (2016)
	Wang	2019	12	•	°	°	•	°	°	°	°	°	°	°	°	°	•	°	°	°	°	•	°	°	°	°	•	°	°	°	°	°	•	Wang et al. (2019)

*List of all references from 2010 to 2020 that used combined EEG-EOG methods. The references are classified by domain of application*.

## Results

**Figure 2:**
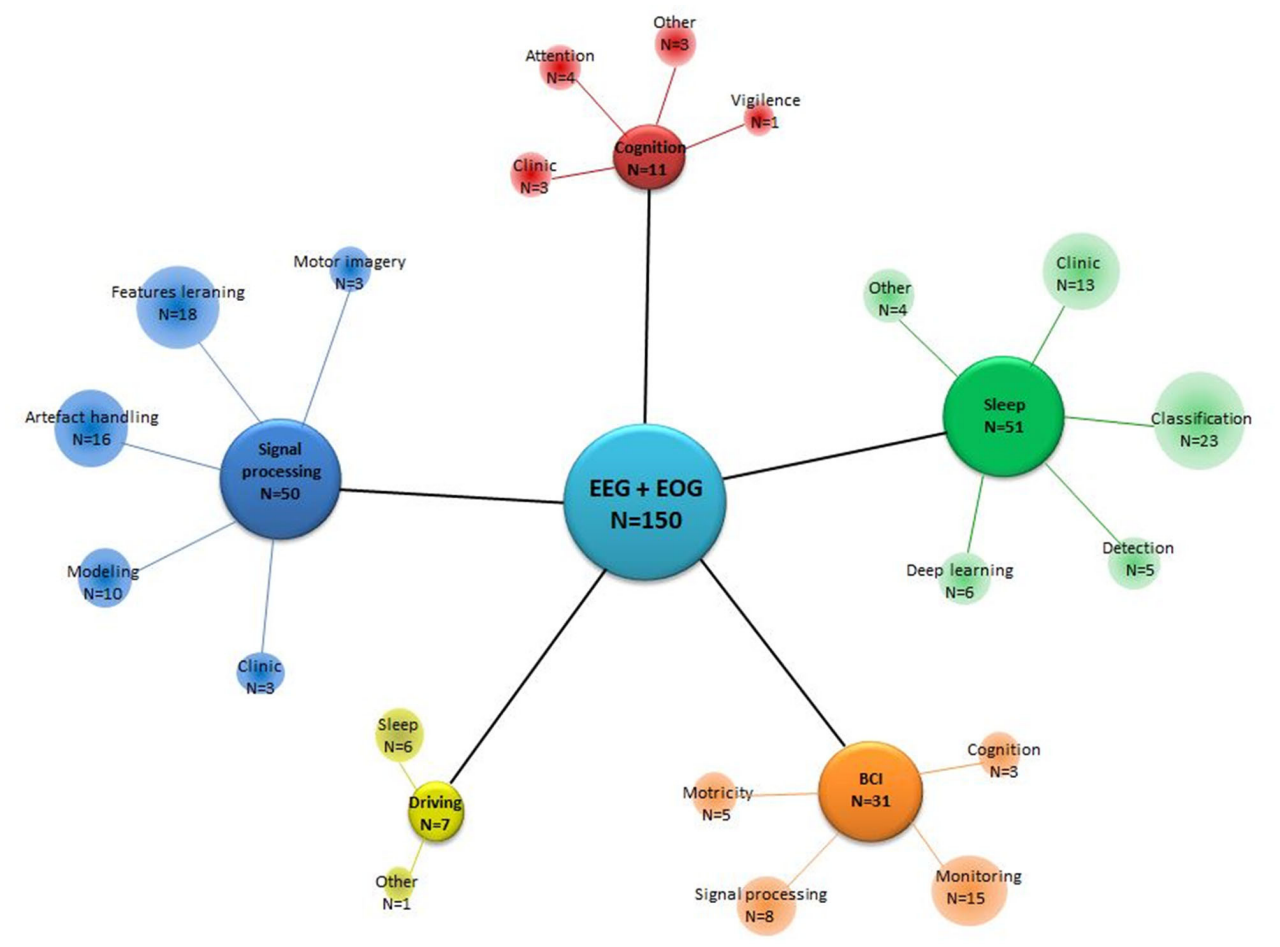
Focus of the studies, gathering papers according to their domain of application. *N*, Number of studies that fit in a category according to the central focus.

A total of 150 papers were selected for inclusion in this review. Our search methodology returned 121 journal papers and 29 conference papers that met our criteria. We noted that the article papers were published in different journals; however, all the conference papers were only published in numerous years of the International Conferences of the Institute of Electrical and Electronics Engineers (IEEE). The included papers have combined EEG-EOG in various domains of application (see Figure 2). Most studies focused notably on sleep, signal processing, and BCI categories, respectively 34, 33, and 21% of the total selected studies. Sleep category concerns EEG and EOG data for sleep classification in healthy and clinic patients as well as using deep learning for classification of sleep stages and disorders. The signal processing category regroups the development of tools, such as analyzing, modifying, and synthesizing signals, handling artifacts, learning features, and training models. BCI category groups the manipulation of hybrid EEG-EOG systems as a valuable communication tool for monitoring computers, wheelchairs, or a robotic exoskeleton. Seven percent of the selected studies belong to the cognition category. They were particularly related to analyzing the performance of attention and cognition. The remaining papers (5%) explored different analyses of driving with conditions such as sleep deprivation or fatigue.

**Figure 3:**
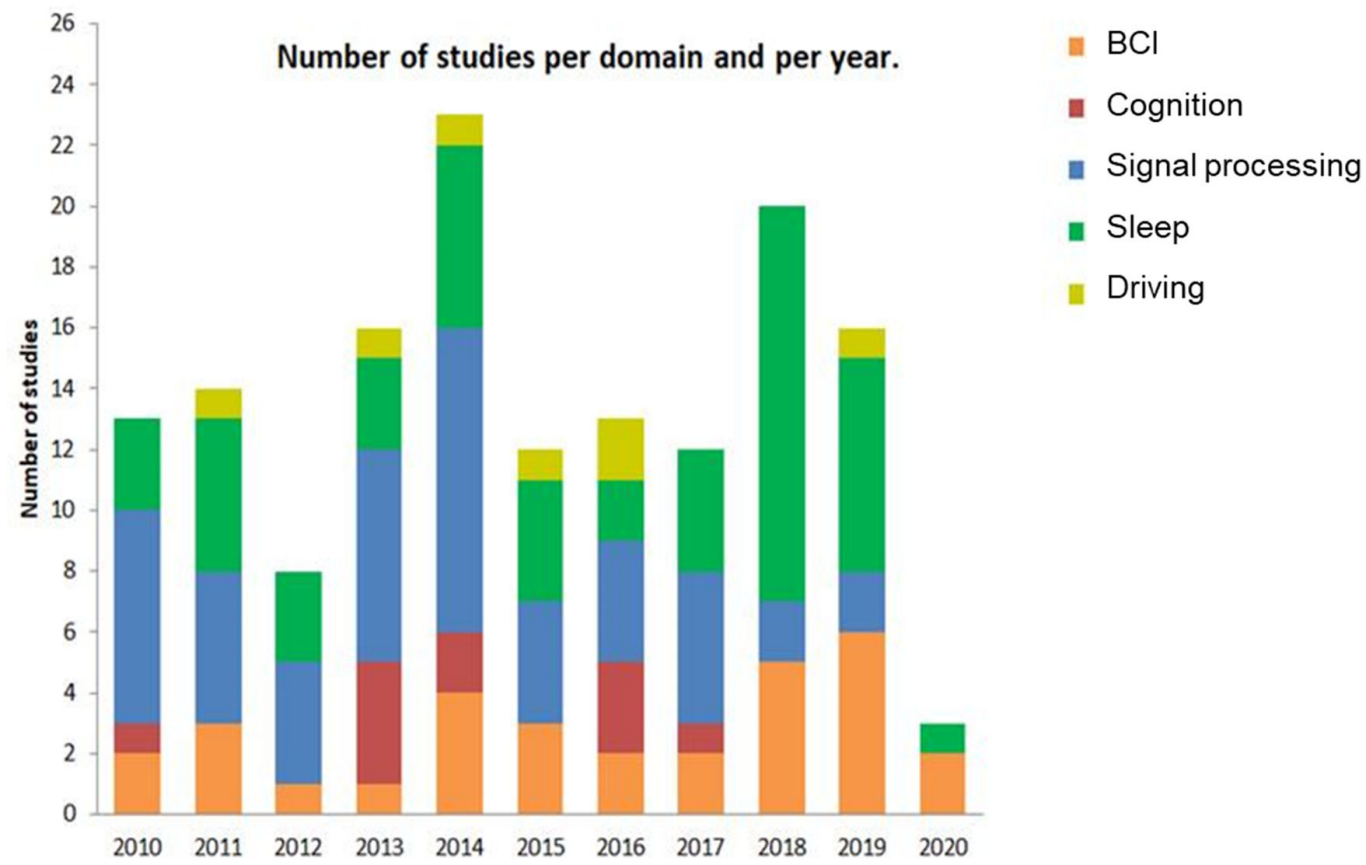
The number of publications for each domain of application per year.

Figure 3 indicates the evolution of EEG-EOG investigations since 2010 in each domain of application. We did not observe any clear tendency apart from a growing concern for BCI. The first 3 months of 2020 associated with the years 2019 and 2018 alone account for 42% of the total selected publications. Nevertheless, given the relatively small number of publications to date, it is too early to draw conclusions about trends.

**Figure 4:**
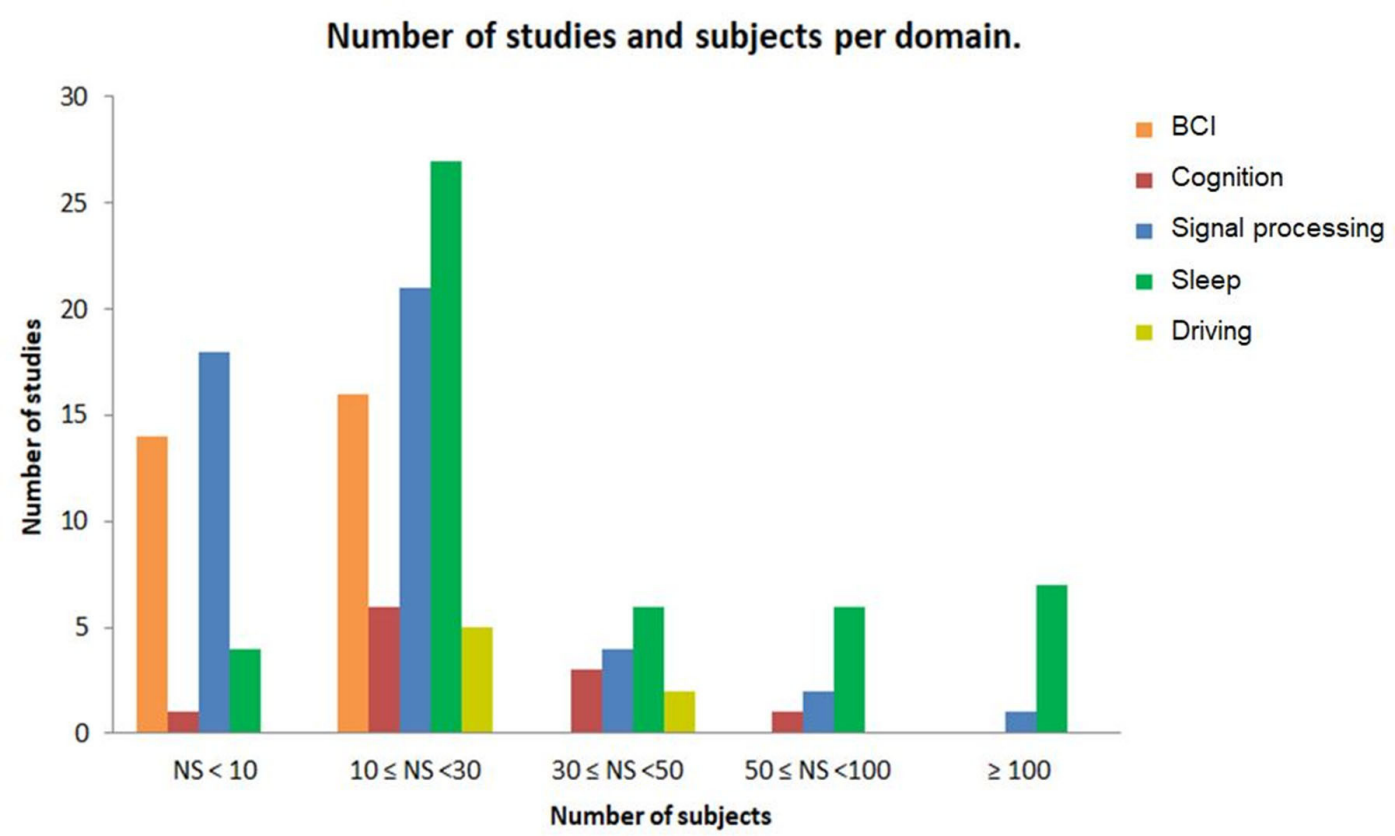
The number of studies and subjects per domain of application. Each bar represents the number of studies and the number of subjects for each domain in database of this review.

The number of subjects included in each study varies expressively across the different domains of application (see Figure 4). Seventy-five percent of the included datasets contained fewer than 30 participants. Some studies have datasets with a higher number of participants with at least 600 subjects (Singh et al., 2014; Olsen et al., 2017; Korkalainen et al., 2019), while others included studies used datasets with <5 subjects, particularly in BCI and signal processing categories.

**Figure 5:**
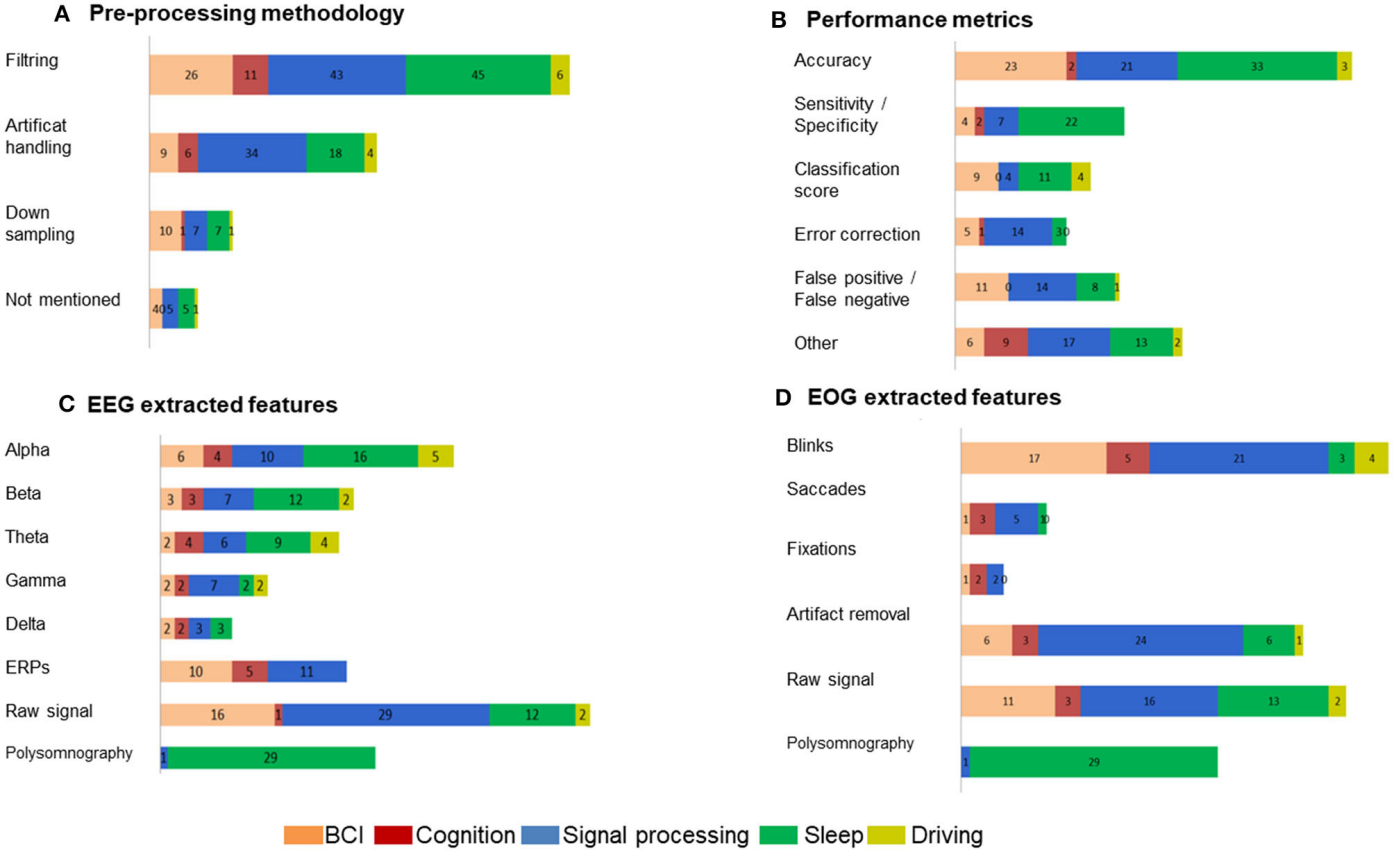
Percentage of the methodology employed in the selected EEG-EOG studies. **(A)** Pre-processing procedures, **(B)** identified performance metrics, **(C)** EEG extracted features, and **(D)** EOG extracted features.

**Figure 6:**
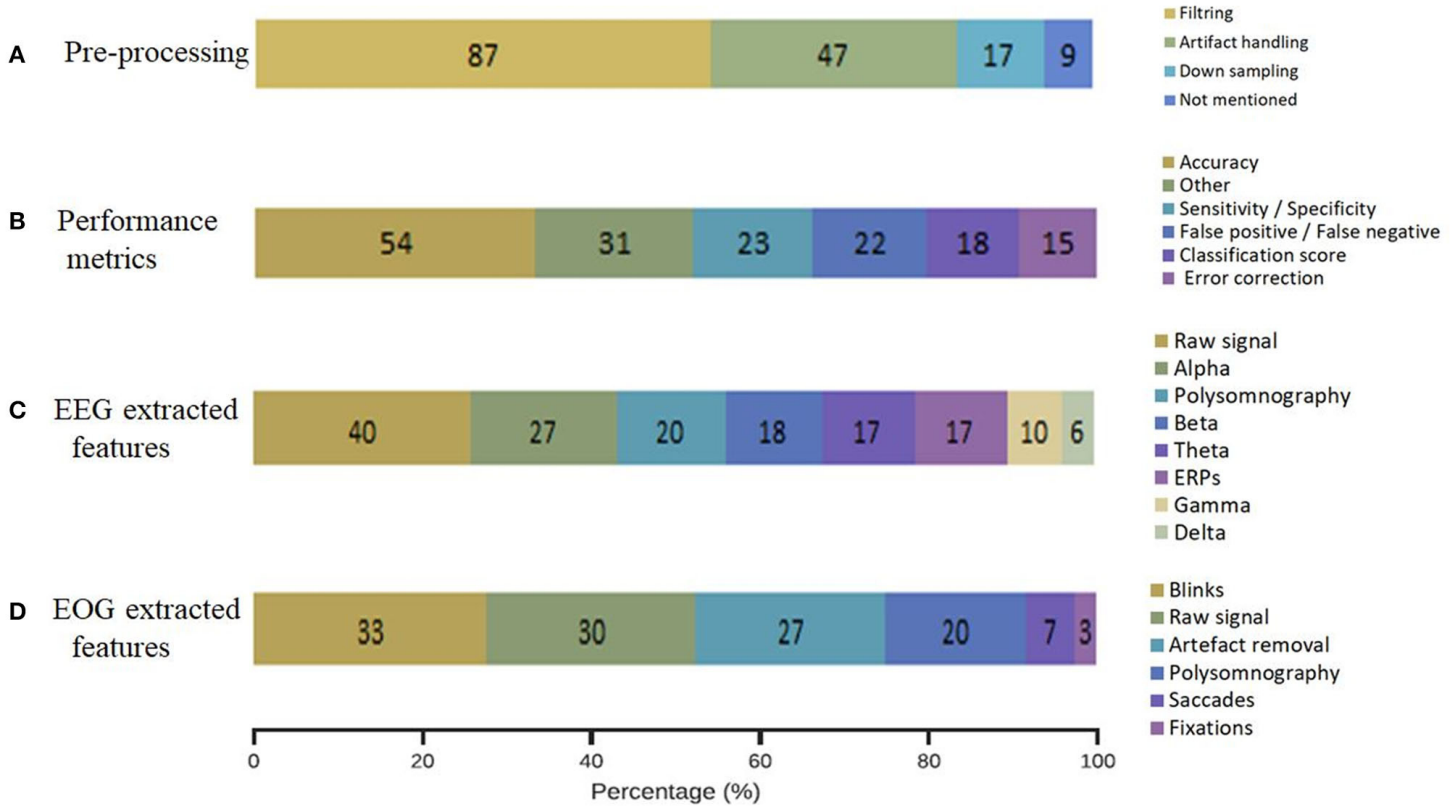
Methodology applied choices in each domain of application. **(A)** Pre-processing methodology, **(B)** performance metrics, **(C)** EEG extracted features, **(D)** EOG extracted features.

When reviewing our included studies, we regrouped some of the common pre-processing steps employed (Figure 5A). The pre-processing methodology presented some routine steps, such as bandpass filtering, downsampling, windowing, interpolating the bad channels, computing the average reference, or removing line noise. Eighty-seven percent of the selected studies used band-pass filters and notch filters that allow the extraction of characteristic signals located in the stimulus frequency and that remove noise and artifacts. Forty-seven percent of studies describe artifact handling, which consisted of eliminating certain types of noise, such as eyes and muscle artifacts. Seventeen percent of the studies downsampled the signal acquired at a higher sampling rate to 256 Hz or less. Even when deleting the noise might be essential to achieving relevant EEG decoding efficiency, 9% of the included studies did not explicitly mention pre-processing steps. The distribution of the selected pre-processing steps according to each domain of application is shown in Figure 6A. In particular, the filtering process was employed by studies in BCI (*n* = 26), sleep (*n* = 43), and signal processing (*n* = 45). Artifact handling was mostly used in signal processing (*n* = 34) and sleep (*n* = 18) domains. Downsampling was used in BCI, sleep (*n* = 7), and signal processing (*n* = 7) categories.

When assessing the performance of signal processing in the selected studies (Figure 5B), we were not surprised to find that most of them applied common metrics that are derived from confusion matrices. We classified the performance metrics into the component that emerged from the included studies: accuracy estimation (54%), sensitivity and specificity values (23%), false positive and/or false negative detection (22%), classification score (F1-score, Kappa score or Support Vector Machine score) (18%), an approximation of error correction (15%), and other non-common metrics (31%).

The distribution of the selected performance metrics according to each domain of application is shown in Figure 6B. As expected, we noted an important evaluation of accuracy in the BCI (*n* = 23), signal processing (*n* = 21), and sleep (*n* = 33) domains.

Figure 5C details the performed analysis on the registered EEG signals from the included studies. The evaluation revealed the following extracted features: raw signal (40%), alpha frequency (27%), polysomnography (20%), beta frequency (18%), theta frequency (17%), ERPs (17%), gamma frequency (10%), and delta frequency (6%).

The distribution of the EEG extracted features according to each domain of application is shown in Figure 6C. Particularly, alpha, beta, and theta frequencies were investigated in sleep and signal processing domains. ERPs were analyzed in BCI (*n* = 10), cognition (*n* = 5), and signal processing domains (*n* = 11). The raw signal was used in the majority of the domains BCI (*n* = 16), signal processing (*n* = 29), and sleep (*n* = 12). As expected, polysomnography was exanimated principally in sleep studies (*n* = 29).

The classification of the extracted features from the registered EOG signals showed the following results: blinks (33%), raw signal (30%), artifact removal (27%), polysomnography (20%), saccades (7%) and fixation (3%) (Figure 5D). The analysis of blinks movements was particularly present in BCI (*n* = 17) and algorithms (*n* = 21) studies. The artifact removal was majority used in algorithms (*n* = 24) and equally used in BCI and sleep (*n* = 6). The raw signal was analyzed in BCI, (*n* = 11), algorithms (*n* = 16) and sleep (*n* = 13).

## Discussion

The objective of this work is to present a comprehensive summarized review of combined EEG-EOG studies and a taxonomy model of the last decade. Our taxonomy model presents an overview of classification by domains of application: sleep, BCI, signal processing, cognition, and driving. It is worth mentioning that these identified categories are significantly relevant to research in the exploration of human factors in the aeronautical industry and neural engineering. Figures 2–5 present the distribution of combined EEG-EOG papers of the taxonomy according to domains of application, while Figure 6 illustrates the different methodological approaches. The motivation behind this is to derive trends about how each feature type and domain of application have been investigated. This may assist researchers in exploring human factors and the identification of feature types in future combined EEG-EOG work. Here, we discuss the most relevant outcomes from our results section and review the involvement of the different findings highlighted above in aeronautics. We also provide recommendations for combined EEG-EOG studies to facilitate extension in the field. Finally, we present some limitations in our work and future perspectives.

Sleep and fatigue prediction are the main challenges explored in investigations on human factors. In this context, one of the outcomes of this review was the identification of EEG and EOG features that may help to detect unintentional sleep in airline pilots. Our results highlight relevant features that help to study aspects related to the sleep of pilots, aiming to improve safety conditions. For example, typical markers of sleep are the disappearance of the alpha rhythm and the appearance of roving eye movements. Fatigue, irregular, and long working hours, and time zone crossings can change sleep-wake cycles, alert levels, and affect pilot decisions during a flight. These issues cause excessive sleepiness, unintentional sleep, and increase the risk of accidents. Some investigations with airline pilots observed that this working environment typically exposes several sleep and health issues, particularly extreme sleepiness, unintentional naps, and fatigue (Petrie and Dawson, 1997; Wright and McGown, 2001). However, such studies are still incipient, and knowing the characteristics of sleep prediction is necessary in supporting safety policies and working practices. The tasks performed by pilots are complex and involve several functions, including cognitive, technical, and relationship skills. They involve concentration, the ability to work under pressure, adaptation to operational modifications, teamwork, the prediction of the consequences, the interpretation of signals, and quick decisions (Itani, 2009). When excessive sleepiness occurs, such skills may be impaired, thereby affecting flight safety. Ingre et al. (2014) have observed that unintentional sleep in pilots may compromise the safety of flights. Therefore, the ability to observe and predict fatigue provides a significant benefit in avoiding incidents and accidents.

Fatigue and sleepiness decrease task-related activity in the frontal and parietal regions and also reduce activity in and connectivity with, the extrastriate visual cortex during tasks that require visuospatial attention (Chee, 2010). These neural changes affect the behavior of pilots, for example, they might miss specific visual or auditory stimulus. This disturbance alternates the top-down allocation of attentional resources, such as the attentional orientation of a target. Sleep disorders during flights impair sustained attention because of the decreased activity of the dorsolateral prefrontal cortex and parietal sulcus (Kong and Soon, 2012). According to these neural alterations, our review suggests that combined EEG-EOG analysis may provide useful information in evaluating sleep and sleep-related disorders in pilots during flights. Future research could focus on improving EOG models and conduct a comprehensive evaluation of the content of various EOG results, studying the proportion of slow eye movement, rapid eye movements, and no eye movements, the time distribution, and mixtures of slow/fast/no eye movements.

Figure 3 revealed the distribution of combined EEG-EOG works each year since 2010. Although the absolute number of combined EEG-EOG studies is relatively smaller compared to other single EEG (Bonanni et al., 2016) or eye movement applications (Hodgson et al., 2019), there is an important amount of interest in combined EEG-EOG studies. Unexpectedly, we were unable to draw conclusions about trends in the number of studies. We only observed that the first 3 months of 2020 associated with the years 2019 and 2018 alone account for 42% of total BCI publications. Due to the relatively small number of publications to date, it is too early to make assumptions about trends, but a possible explanation for this could be that BCI technology is rapidly gaining attention from scientists, engineers, clinicians, and the general public. The initial applications of BCI aimed to rehabilitate patients with neuromuscular diseases such as amyotrophic lateral sclerosis and injuries to the spinal cord. Here, we observed various patterns in BCI techniques, including EEG based spelling system (Lee et al., 2018), control of cursors (He et al., 2020), robotic arms (Zhang et al., 2019b), prostheses (Soekadar et al., 2015), wheelchairs (Huang et al., 2019), and other complex devices.

In aeronautics, passive BCIs have been successfully used to detect and characterize several operator mental states such as workload and fatigue (Zander et al., 2010; Khan and Hong, 2015; Roy and Frey, 2016). Functional Near-InfraRed Spectroscopy (fNIRS) connectivity based on passive BCI metrics has been explored to detect a pilot's engagement when undertaking automated and manual landing scenarios (Verdière et al., 2018). Analysis confirmed that these two situations contrast, as manual landing led to significantly higher subjective NASA-TLX scores than automated landing. Designing a system capable of measuring continuous monitoring based on eyes and brain signal features or detecting an operator's degraded states would enhance both safety and performance. The achievements of BCI tend to be that it improves aeronautic performance in time-critical situations by decoding an operator's neural activity associated with the act. As brain activity precedes motor performance (Belkhiria et al., 2019), the decoded output in real time could ameliorate the operator's action. Our review showed that some EEG-EOG studies used online BCI to detect error-related potentials and reduce error-rate, improving overall performance. While these methods are promising, they have not been tested in aeronautics. An interesting study (Callan et al., 2016) used magnetoencephalography (MEG) and BCI to explore neuroadaptive automation to reduce a pilot's time response to a hazardous event by decoding their perceptual-motor intentions. The BCI system succeeded in decoding motor intention faster than manual control in response to a change in attitude while ignoring ongoing motor and visual induced activity related to piloting the airplane. While this system used 400 channels, the authors expressed the possibility of making a dedicated real-time system working with mobile EEG. Such signal processing handling methods could separate artifacts from brain-related activity in flight even in an open cockpit biplane (Callan et al., 2016). In the future, research in combined EEG-EOG systems could use a BCI-decoder to distinguish between brain activity that responds to changes in the visual field and motor intention in a flight simulator or even in real aircraft.

Our analysis shows that the number of subjects included in each study varies across the different domains of application. It should also be highlighted that database availability is different from one domain to another. We noted that the most important datasets come from clinical investigations of conditions such as epilepsy, Parkinson's Disease, and sleep disorders. However, in other fields with more exploratory goals, studies rely on data registered in laboratory settings with a limited number of participants (e.g., 30 participants). Some studies explained that the reduced number of subjects is due to experimental conditions, time, and device limitations (Al-Hudhud et al., 2019). Further studies should use more strict inclusion and exclusion criteria for their datasets. This may generate a more robust statistical result.

Figures 5, 6 show methodologies of data pre-processing, various extracted features from EEG and EOG signals, and performance metrics. The literature shows that performance metrics, including accuracies classification, determine computer-based analysis in various applications (Acharya et al., 2011). The objective is to construct algorithms that overcome recognized methods (Faust et al., 2010). Here, most of the selected studies used an offline or an online system. Commonly, the system involves three consecutive processing steps: (i) pre-processing (Kalayci and Özdamar, 1995; Rao and Derakhshani, 2005); (ii) feature extraction (Tyagi and Nehra, 2018); and (iii) classification (Wang et al., 2014b). All of the selected studies explored at least one parameter from the processing pipeline steps (Table 1). Recent advances in signal processing analysis provide a powerful tool for modeling complex probability distributions by automatically discovering intermediate abstractions from a huge amount of basic features. Deep machine learning and artificial intelligence have shown great promise in helping make sense of EEG signals due to their capacity to learn good feature representations from raw data (Dehais et al., 2018). In that sense, the number of aeronautics publications applying these techniques to EEG signal processing has seen an exponential increase in recent years. The increasing interest in methodologies for processing EEG-EOG signal data (e.g., deep learning, machine learning, and artificial intelligence) in relation to human factors noticeably reflects an emergent interest in these kinds of approaches. Research in aeronautic sensors and signal processing systems (e.g., EEG and EOG) is exploring revolutionary improvements, potentially enhancing civil and military applications in fighter planes, helicopters, and the remote operation of drones. Yet another trend in sensor and signal processing involves blending artificial intelligence and machine learning into system designs and the incorporation of electrophysiological sensors and associated signal processing methods into equipment, such as aviation headsets, may enhance operational safety (Wilson et al., 2020).

## Limitations and Conclusions

The review presented state-of-the-art research on the characteristics and applications of both EEG and EOG signals in aeronautics. Our taxonomy and statistical analysis described a number of operational mechanisms, such as extracted features, pre-processing treatment, and performance metrics. We discussed how these methodologies could be adopted by researchers examining human factors and aeronautics. The effectiveness of combining EEG and EOG as a psycho-physiological tool is unequivocal. However, numerous challenges still need to be resolved. An exciting and unprecedented approach would be the assessment of both eye movement and brain activity during novel paradigms using dry electrodes that are integrated into existing control and communication peripherals. An equipped headset measuring real-time EEG and EOG may pose a great challenge in terms of applicability and generalizability to both commercial and scientific research into mobile EEG devices. This method would improve human-system interaction by making it possible to follow up on data about eye movements and use it to determine the psycho-physiological state of a person. Studies combining both EEG and EOG technologies and a review of the application of these in several fields, including in laboratory and real-world situations, are of particular value, as these technologies could be of interest in areas using both visual and auditory information simultaneously through headphones and gaze inputs, including in aeronautics, helicopters, teleoperation drones, naval systems, and control-command centers.

Despite the clear value of data collected and presented here, this review has some limitations. First, despite the use of a well-founded methodology to identify pertinent studies on the topic; the review did not cover all existing papers. It is also important to stress the error risk that could occur as a result of the inclusion methodology. Even though we examined 150 studies among a wider pool of 255 from Pubmed research, this is an exhaustive list nor should we undervalue the relevance of the studies that were not included. Second, in managing the length of the review, we limited our analysis to the main domains of applications. However, some studies could involve two or more application domains. Some topics could overlap, such as BCI and signal processing (Daly et al., 2015; Ivorra et al., 2018). We did consider categorizing another field of application related to clinical applications. However, this would have moved the focus away from aeronautics. Finally, as with any literature review, new articles are being published and new trends are being established and future studies should eventually be added to the analysis.

## Author Contributions

CB: conceptualization, methodology, data curation, and writing-original draft preparation. VP: conceptualization, methodology, writing, reviewing, and editing. Both authors contributed to the article and approved the submitted version.

## Conflict of Interest

The authors declare that the research was conducted in the absence of any commercial or financial relationships that could be construed as a potential conflict of interest.

## References

[B1] Åkerstedt T. Hallvig D. Anund A. Fors C. Schwarz J. Kecklund G. (2013). Having to stop driving at night because of dangerous sleepiness - awareness, physiology and behaviour. J. Sleep Res. 22, 380–388. 10.1111/jsr.12042 23509866

[B2] Acharya U. R. Sree S. V. Chattopadhyay S. Yu W. Ang P. C. A. (2011). Application of recurrence quantification analysis for the automated identification of epileptic EEG signals. Int. J. Neural Syst. 21, 199–211. 10.1142/S0129065711002808 21656923

[B3] Acuna O. V. Aqueveque P. Pino E. J. (2014). Eye-tracking capabilities of low-cost EOG system, in 2014 36th Annual International Conference of the IEEE Engineering in Medicine and Biology Society (Chicago, IL: IEEE), 610–613. 10.1109/EMBC.2014.6943665 25570033

[B4] Ahn S. Nguyen T. Jang H. Kim J. G. Jun S. C. (2016). Exploring neuro-physiological correlates of drivers' mental fatigue caused by sleep deprivation using simultaneous EEG, ECG, and fNIRS data. Front. Hum. Neurosci. 10:219. 10.3389/fnhum.2016.00219 27242483 PMC4865510

[B5] Al-Hudhud G. Alqahtani L. Albaity H. Alsaeed D. Al-Turaiki I. (2019). Analyzing passive BCI signals to control adaptive automation devices. Sensors 19:3042. 10.3390/s19143042 31295908 PMC6678787

[B6] Alvarez T. L. Semmlow J. L. Pedrono C. (2005). Divergence eye movements are dependent on initial stimulus position. Vision Res. 45, 1847–1855. 10.1016/j.visres.2005.01.017 15797774

[B7] Anderson I. H. (1937). Studies in the eye movements of good and poor readers. Psychol. Monogr. 48, 1–35. 10.1037/h0093391

[B8] Andreotti F. Phan H. Cooray N. Lo C. Hu M. T. M. De Vos M. (2018). Multichannel sleep stage classification and transfer learning using convolutional neural networks, in Proceedings of the Annual International Conference of the IEEE Engineering in Medicine and Biology Society, EMBS (Honolulu, HI: Institute of Electrical and Electronics Engineers Inc.), 171–174. 10.1109/EMBC.2018.8512214 30440365

[B9] Arnin J. Anopas D. Horapong M. Triponyuwasi P. Yamsa-Ard T. Iampetch S.. (2013). Wireless-based portable EEG-EOG monitoring for real time drowsiness detection, in Proceedings of the Annual International Conference of the IEEE Engineering in Medicine and Biology Society, EMBS (Osaka: Conf Proc IEEE Eng Med Biol Soc), 4977–4980. 10.1109/EMBC.2013.6610665 24110852

[B10] Arnin J. Kahani D. Lakany H. Conway B. A. (2018). Evaluation of different signal processing methods in time and frequency domain for brain-computer interface applications, in Proceedings of the Annual International Conference of the IEEE Engineering in Medicine and Biology Society, EMBS (Honolulu, HI: Institute of Electrical and Electronics Engineers Inc.), 235–238. 10.1109/EMBC.2018.8512193 30440381

[B11] Arslan Tuncer S. Kaya T. (2018). True random number generation from bioelectrical and physical signals. Comput. Math. Methods Med. 2018:3579275. 10.1155/2018/3579275 30065779 PMC6051287

[B12] Assi E. B. Rihana S. Sawan M. (2014). Kmeans-ICA based automatic method for ocular artifacts removal in a motorimagery classification, in 2014 36th Annual International Conference of the IEEE Engineering in Medicine and Biology Society, EMBC 2014 (Chicago, IL: Institute of Electrical and Electronics Engineers Inc.), 6655–6658. 10.1109/EMBC.2014.6945154 25571522

[B13] Aziz F. Arof H. Mokhtar N. Mubin M. (2014). HMM based automated wheelchair navigation using EOG traces in EEG. J. Neural Eng. 11:056018. 10.1088/1741-2560/11/5/056018 25188730

[B14] Babiloni C. Barry R. J. Başar E. Blinowska K. J. Cichocki A. Drinkenburg W. H. I. M.. (2020). International federation of clinical neurophysiology (IFCN) – EEG research workgroup: recommendations on frequency and topographic analysis of resting state EEG rhythms. Part 1: applications in clinical research studies. Clin. Neurophysiol. 131, 285–307. 10.1016/j.clinph.2019.06.234 31501011

[B15] Babiloni C. Vecchio F. Infarinato F. Buffo P. Marzano N. Spada D.. (2011). Simultaneous recording of electroencephalographic data in musicians playing in ensemble. Cortex 47, 1082–1090. 10.1016/j.cortex.2011.05.006 21664610

[B16] Badesa F. J. Diez J. A. Catalan J. M. Trigili E. Cordella F. Nann M.. (2019). Physiological responses during hybrid BNCI control of an upper-limb exoskeleton. Sensors 19:4931. 10.3390/s19224931 31726745 PMC6891352

[B17] Bai Y. Wan X. Zeng K. Ni Y. Qiu L. Li X. (2016). Reduction hybrid artifacts of EMG-EOG in electroencephalography evoked by prefrontal transcranial magnetic stimulation. J. Neural Eng. 13:066016. 10.1088/1741-2560/13/6/066016 27788128

[B18] Barea R. Boquete L. Mazo M. López E. (2002). Wheelchair guidance strategies using EOG. J. Intell. Robot. Syst. Theory Appl. 34, 279–299. 10.1023/A:101635950379612611358

[B19] Barry R. J. De Blasio F. M. (2017). EEG differences between eyes-closed and eyes-open resting remain in healthy ageing. Biol. Psychol. 129, 293–304. 10.1016/j.biopsycho.2017.09.010 28943465

[B20] Barry R. J. De Blasio F. M. Bernat E. M. Steiner G. Z. (2015). Event-related EEG time-frequency PCA and the orienting reflex to auditory stimuli. Psychophysiology 52, 555–561. 10.1111/psyp.12376 25353309

[B21] Barthélemy Q. Mayaud L. Renard Y. Kim D. Kang S. W. Gunkelman J.. (2017). Online denoising of eye-blinks in electroencephalography. Neurophysiol. Clin. 47, 371–391. 10.1016/j.neucli.2017.10.059 29169769

[B22] Behrens F. MacKeben M. Schröder-Preikschat W. (2010). An improved algorithm for automatic detection of saccades in eye movement data and for calculating saccade parameters. Behav. Res. Methods 42, 701–708. 10.3758/BRM.42.3.701 20805592

[B23] Belkhiria C. Mssedi E. Habas C. Driss T. de Marco G. (2019). Collaboration of cerebello-rubral and cerebello-striatal loops in a motor preparation task. Cerebellum 18, 203–211. 10.1007/s12311-018-0980-z 30276521

[B24] Bernhardt K. A. Poltavski D. Petros T. Ferraro F. R. Jorgenson T. Carlson C.. (2019). The effects of dynamic workload and experience on commercially available EEG cognitive state metrics in a high-fidelity air traffic control environment. Appl. Ergon. 77, 83–91. 10.1016/j.apergo.2019.01.008 30832781

[B25] Binnie C. D. Dekker E. Smit A. Van Der Linden G. (1982). Practical considerations in the positioning of EEG electrodes. Electroencephalogr. Clin. Neurophysiol. 53, 453–458. 10.1016/0013-4694(82)90010-4 6175507

[B26] Bizopoulos P. A. Al-Ani T. Tsalikakis D. G. Tzallas A. T. Koutsouris D. D. Fotiadis D. I. (2013). An automatic electroencephalography blinking artefact detection and removal method based on template matching and ensemble empirical mode decomposition, in Proceedings of the Annual International Conference of the IEEE Engineering in Medicine and Biology Society (EMBS) (Osaka), 5853–5856. 10.1109/EMBC.2013.6610883 24111070

[B27] Bleichner M. G. Debener S. (2017). Concealed, unobtrusive ear-centered EEG acquisition: cEEGrids for transparent EEG. Front. Hum. Neurosci. 11:163. 10.3389/fnhum.2017.00163 28439233 PMC5383730

[B28] Bonanni L. Franciotti R. Nobili F. Kramberger M. G. Taylor J. P. Garcia-Ptacek S.. (2016). EEG markers of dementia with lewy bodies: a multicenter cohort study. J. Alzheimer's Dis. 54, 1649–1657. 10.3233/JAD-160435 27589528

[B29] Borghini G. Aricò P. Flumeri G. Cartocci G. Colosimo A. Bonelli S.. (2017). EEG-based cognitive control behaviour assessment: an ecological study with professional air traffic controllers. Sci. Rep. 7:547. 10.1038/s41598-017-00633-7 28373684 PMC5428823

[B30] Borghini G. Astolfi L. Vecchiato G. Mattia D. Babiloni F. (2014). Measuring neurophysiological signals in aircraft pilots and car drivers for the assessment of mental workload, fatigue and drowsiness. Neurosci. Biobehav. Rev. 44, 58–75. 10.1016/j.neubiorev.2012.10.003 23116991

[B31] Brams S. Hooge I. T. C. Ziv G. Dauwe S. Evens K. De Wolf T.. (2018). Does effective gaze behavior lead to enhanced performance in a complex error-detection cockpit task? PLoS ONE 13:e0207439. 10.1371/journal.pone.0207439 30462695 PMC6248957

[B32] Breitwieser C. Neuper C. Müller-Putz G. R. (2011). A concept to standardize raw biosignal transmission for brain-computer interfaces, in Proceedings of the Annual International Conference of the IEEE Engineering in Medicine and Biology Society, EMBS (Boston, MA: Conf Proc IEEE Eng Med Biol Soc), 6377–6380. 10.1109/IEMBS.2011.6091574 22255797

[B33] Brown L. Van De Molengraft J. Yazicioglu R. F. Torfs T. Penders J. Van Hoof C. (2010). A low-power, wireless, 8-channel EEG monitoring headset, in 2010 Annual International Conference of the IEEE Engineering in Medicine and Biology Society, EMBC'10 (Buenos Aires), 4197–4200. 10.1109/IEMBS.2010.5627393 21096892

[B34] Bulling A. Roggen D. Tröster G. (2009). Wearable EOG goggles: seamless sensing and context-awareness in everyday environments. J. Ambient Intell. Smart Environ. 1, 157–171. 10.3233/AIS-2009-0020

[B35] Caldwell J. A. Hall K. K. Erickson B. S. (2002). EEG data collected from helicopter pilots in flight are sufficiently sensitive to detect increased fatigue from sleep deprivation. Int. J. Aviat. Psychol. 12, 19–32. 10.1207/S15327108IJAP1201_3

[B36] Callan D. E. Durantin G. Terzibas C. (2015). Classification of single-trial auditory events using dry-wireless EEG during real and motion simulated flight. Front. Syst. Neurosci. 9:11. 10.3389/fnsys.2015.00011 25741249 PMC4330719

[B37] Callan D. E. Terzibas C. Cassel D. B. Sato M. A. Parasuraman R. (2016). The brain is faster than the hand in split-second intentions to respond to an impending hazard: a simulation of neuroadaptive automation to speed recovery to perturbation in flight attitude. Front. Hum. Neurosci. 10:187. 10.3389/fnhum.2016.00187 27199710 PMC4846799

[B38] Camfferman D. Kennedy J. D. Gold M. Simpson C. Lushington K. (2013). Sleep and neurocognitive functioning in children with eczema. Int. J. Psychophysiol. 89, 265–272. 10.1016/j.ijpsycho.2013.01.006 23353660

[B39] Cannon J. Krokhmal P. A. Chen Y. Murphey R. (2012). Detection of temporal changes in psychophysiological data using statistical process control methods. Comput. Methods Programs Biomed. 107, 367–381. 10.1016/j.cmpb.2011.01.003 21377752

[B40] Carl C. Açik A. König P. Engel A. K. Hipp J. F. (2012). The saccadic spike artifact in MEG. Neuroimage 59, 1657–1667. 10.1016/j.neuroimage.2011.09.020 21963912

[B41] Carl W. Sem-Jacobsen M. D. Nilseng O. Patten C. Eriksen O. (1959). Electroencephalographic recording in simulated combat flight in a jet fighter plane. The pilot's level of consciousness. Electroencephalogr. Clin. Neurophysiol. 11, 154–155. 10.1016/0013-4694(59)90018-5 13630243

[B42] Casson A. J. Rodriguez-Villegas E. (2012). Signal agnostic compressive sensing for body area networks: comparison of signal reconstructions, in Proceedings of the Annual International Conference of the IEEE Engineering in Medicine and Biology Society, EMBS (San Diego, CA: Conf Proc IEEE Eng Med Biol Soc), 4497–4500. 10.1109/EMBC.2012.6346966 23366927

[B43] Chan H. L. Tsai Y. T. Meng L. F. Wu T. (2010). The removal of ocular artifacts from EEG signals using adaptive filters based on ocular source components. Ann. Biomed. Eng. 38, 3489–3499. 10.1007/s10439-010-0087-2 20532631

[B44] Chang W.-D. (2019). Electrooculograms for human–computer interaction: a review. Sensors 19:2690. 10.3390/s19122690 31207949 PMC6630230

[B45] Chang W. D. Cha H. S. Im C. H. (2016). Removing the interdependency between horizontal and vertical eye-movement components in electrooculograms. Sensors 16:227. 10.3390/s16020227 26907271 PMC4801603

[B47] Charbonnier S. Zoubek L. Lesecq S. Chapotot F. (2011). Self-evaluated automatic classifier as a decision-support tool for sleep/wake staging. Comput. Biol. Med. 41, 380–389. 10.1016/j.compbiomed.2011.04.001 21497802

[B48] Chee M. T. J. (2010). Lapsing when sleep deprived: neural activation characteristics of resistant and vulnerable individuals. Neuroimage 51, 835–843. 10.1016/j.neuroimage.2010.02.031 20171288

[B49] Chen Y.-H. de Beeck M. Vanderheyden L. Carrette E. Mihajlović V. Vanstreels K.. (2014). Soft, comfortable polymer dry electrodes for high quality ECG and EEG recording. Sensors 14, 23758–23780. 10.3390/s141223758 25513825 PMC4299086

[B50] Cho H. Kwon M. Jang H. Lee J. B. Yoon K. C. Jun S. C. (2016). Herbal extracts that reduce ocular oxidative stress may enhance attentive performance in humans. Comput. Intell. Neurosci. 2016:4292145. 10.1155/2016/4292145 28090203 PMC5206474

[B51] Christensen J. A. E. Frandsen R. Kempfner J. Arvastson L. Christensen S. R. Jennum P.. (2012). Separation of Parkinson's patients in early and mature stages from control subjects using one EOG channel, in Proceedings of the Annual International Conference of the IEEE Engineering in Medicine and Biology Society, EMBS (San Diego, CA: Conf Proc IEEE Eng Med Biol Soc), 2941–2944. 10.1109/EMBC.2012.6346580 23366541

[B52] Christensen J. A. E. Zoetmulder M. Koch H. Frandsen R. Arvastson L. Christensen S. R.. (2014). Data-driven modeling of sleep EEG and EOG reveals characteristics indicative of pre-parkinson's and parkinson's disease. J. Neurosci. Methods 235, 262–276. 10.1016/j.jneumeth.2014.07.014 25088694

[B53] Cooray N. Andreotti F. Lo C. Symmonds M. Hu M. T. M. De Vos M. (2019). Detection of REM sleep behaviour disorder by automated polysomnography analysis. Clin. Neurophysiol. 130, 505–514. 10.1016/j.clinph.2019.01.011 30772763

[B54] Crea S. Nann M. Trigili E. Cordella F. Baldoni A. Badesa F. J.. (2018). Feasibility and safety of shared EEG/EOG and vision-guided autonomous whole-arm exoskeleton control to perform activities of daily living. Sci. Rep. 8:10823. 10.1038/s41598-018-29091-5 30018334 PMC6050229

[B55] Dahlstrom N. Nahlinder S. (2009). Mental workload in aircraft and simulator during basic civil aviation training. Int. J. Aviat. Psychol. 19, 309–325. 10.1080/10508410903187547

[B56] Daly I. Scherer R. Billinger M. Müller-Putz G. (2015). FORCe: fully online and automated artifact removal for brain-computer interfacing. IEEE Trans. Neural Syst. Rehabil. Eng. 23, 725–736. 10.1109/TNSRE.2014.2346621 25134085

[B57] De Vico Fallani F. Toppi J. Di Lanzo C. Vecchiato G. Astolfi L. Borghini G.. (2012). Redundancy in functional brain connectivity from eeg recordings, in International Journal of Bifurcation and Chaos (World Scientific Publishing Co Pte Ltd). 10.1142/S0218127412501581

[B58] Debener S. Emkes R. De Vos M. Bleichner M. (2015). Unobtrusive ambulatory EEG using a smartphone and flexible printed electrodes around the ear. Sci. Rep. 5:16743. 10.1038/srep16743 26572314 PMC4648079

[B59] Dehais F. Duprès A. Blum S. Drougard N. Scannella S. Roy R. N.. (2019). Monitoring pilot's mental workload using erps and spectral power with a six-dry-electrode EEG system in real flight conditions. Sensors 19:1324. 10.3390/s19061324 30884825 PMC6471557

[B60] Dehais F. Duprès A. Di Flumeri G. Verdière K. J. Borghini G. Babiloni F.. (2018). Monitoring pilot's cognitive fatigue with engagement features in simulated and actual flight conditions using an hybrid fNIRS-EEG passive *BCI*, in 2018 IEEE International Conference on Systems, Man, and Cybernetics (SMC) (Miyazaki: IEEE). 10.1109/SMC.2018.00102

[B61] Dehais F. Roy R. N. Gateau T. Scannella S. (2016). Auditory alarm misperception in the cockpit: an EEG study of inattentional deafness, in Lecture Notes in Computer Science (including subseries Lecture Notes in Artificial Intelligence and Lecture Notes in Bioinformatics) (Toronto, ON: Springer Verlag), 177–187. 10.1007/978-3-319-39955-3_17

[B62] Delisle-Rodriguez D. Villa-Parra A. C. Bastos-Filho T. López-Delis A. Frizera-Neto A. Krishnan S.. (2017). Adaptive spatial filter based on similarity indices to preserve the neural information on EEG signals during on-line processing. Sensors 17:2725. 10.3390/s17122725 29186848 PMC5751387

[B63] Di Flumeri G. Arico P. Borghini G. Colosimo A. Babiloni F. (2016). A new regression-based method for the eye blinks artifacts correction in the EEG signal, without using any EOG channel, in Proceedings of the Annual International Conference of the IEEE Engineering in Medicine and Biology Society (Orlando, FL: EMBS), 3187–3190. 10.1109/EMBC.2016.7591406 28268985

[B64] Di Flumeri G. Aricò P. Borghini G. Sciaraffa N. Di Florio A. Babiloni F. (2019). The dry revolution: evaluation of three different EEG dry electrode types in terms of signal spectral features, mental states classification and usability. Sensors 19:1365. 10.3390/s19061365 30893791 PMC6470960

[B65] Di Nocera F. Camilli M. Terenzi M. (2007). A random glance at the flight deck: pilots' scanning strategies and the real-time assessment of mental workload. J. Cogn. Eng. Decis. Mak. 1, 271–285. 10.1518/155534307X255627

[B66] Di Stasi L. L. McCamy M. B. Martinez-Conde S. Gayles E. Hoare C. Foster M.. (2016). Effects of long and short simulated flights on the saccadic eye movement velocity of aviators. Physiol. Behav. 153, 91–96. 10.1016/j.physbeh.2015.10.024 26597121

[B67] Dimitriadis S. I. Salis C. Linden D. (2018). A novel, fast and efficient single-sensor automatic sleep-stage classification based on complementary cross-frequency coupling estimates. Clin. Neurophysiol. 129, 815–828. 10.1016/j.clinph.2017.12.039 29477981

[B68] Doppelmayr M. Finkenzeller T. Sauseng P. (2008). Frontal midline theta in the pre-shot phase of rifle shooting: differences between experts and novices. Neuropsychologia 46, 1463–1467. 10.1016/j.neuropsychologia.2007.12.026 18280523

[B69] Duchowski A. T. (2017). Eye Tracking Methodology: Theory and Practice. 3rd Edn. Springer International Publishing. 10.1007/978-3-319-57883-5

[B70] Dussault C. Jouanin J.-C. Guezennec C.-Y. (2004). EEG and ECG changes during selected flight sequences. Aviat. Space. Environ. Med. 75, 889–897. 15497370

[B71] Faust O. Acharya U. R. Min L. C. Sputh B. H. C. (2010). Automatic identification of epileptic and background eeg signals using frequency domain parameters. Int. J. Neural Syst. 20, 159–176. 10.1142/S0129065710002334 20411598

[B72] Favre-Felix A. Graversen C. Dau T. Lunner T. (2017). Real-time estimation of eye gaze by in-ear electrodes, in Proceedings of the Annual International Conference of the IEEE Engineering in Medicine and Biology Society, EMBS (Seogwipo: Institute of Electrical and Electronics Engineers Inc.), 4086–4089. 10.1109/EMBC.2017.8037754 29060795

[B73] Fietze I. Penzel T. Partinen M. Sauter J. Küchler G. Suvoro A.. (2015). Actigraphy combined with EEG compared to polysomnography in sleep apnea patients. Physiol. Meas. 36, 385–96. 10.1088/0967-3334/36/3/385 25651914

[B74] Fraiwan L. Lweesy K. Khasawneh N. Wenz H. Dickhaus H. (2012). Automated sleep stage identification system based on time-frequency analysis of a single EEG channel and random forest classifier. Comput. Methods Programs Biomed. 108, 10–19. 10.1016/j.cmpb.2011.11.005 22178068

[B75] Gao J. F. Yang Y. Lin P. Wang P. Zheng C. X. (2010). Automatic removal of eye-movement and blink artifacts from eeg signals. Brain Topogr. 23, 105–114. 10.1007/s10548-009-0131-4 20039116

[B76] Gegenfurtner A. Lehtinen E. Säljö R. (2011). Expertise differences in the comprehension of visualizations: a meta-analysis of eye-tracking research in professional domains. Educ. Psychol. Rev. 23, 523–552. 10.1007/s10648-011-9174-7

[B77] Gevins A. Smith M. E. McEvoy L. K. (2003). EEG and ERP imaging of brain function, in Detection of Change (Boston, MA: Springer US), 133–148. 10.1007/978-1-4615-0294-4_8

[B78] Gharagozlou F. Nasl Saraji G. Mazloumi A. Nahvi A. Motie Nasrabadi A. Rahimi Foroushani A.. (2015). Detecting Driver Mental Fatigue Based on EEG Alpha Power Changes during Simulated Driving. Iran. J. Public Health 44, 1693–1700. 26811821 PMC4724743

[B79] Gharbali A. A. Najdi S. Fonseca J. M. (2018). Investigating the contribution of distance-based features to automatic sleep stage classification. Comput. Biol. Med. 96, 8–23. 10.1016/j.compbiomed.2018.03.001 29529528

[B80] Glos M. Fietze I. Blau A. Baumann G. Penzel T. (2014). Cardiac autonomic modulation and sleepiness: physiological consequences of sleep deprivation due to 40h of prolonged wakefulness. Physiol. Behav. 125, 45–53. 10.1016/j.physbeh.2013.11.011 24291386

[B81] Gordon S. M. Lawhern V. Passaro A. D. McDowell K. (2015). Informed decomposition of electroencephalographic data. J. Neurosci. Methods 256, 41–55. 10.1016/j.jneumeth.2015.08.019 26306657

[B82] Görür D. Halici U. Aydin H. Ongun G. Özgen F. Leblebicioglu K. (2003). Sleep spindles detection using autoregressive modeling, in Proceedings of the International Joint Conference on Neural Networks (Portland, OR).

[B83] Guénolé F. Chevrier É. Stip E. Godbout R. (2014). A microstructural study of sleep instability in drug-naive patients with schizophrenia and healthy controls: sleep spindles, rapid eye movements, and muscle atonia. Schizophr. Res. 155, 31–38. 10.1016/j.schres.2014.03.013 24725849

[B84] Gunnarsdottir K. M. Gamaldo C. Salas R. M. Ewen J. B. Allen R. P. Hu K.. (2020). A novel sleep stage scoring system: combining expert-based features with the generalized linear model. J. Sleep Res. 29:e12991. 10.1111/jsr.12991 32030843 PMC7415500

[B85] Gunnarsdottir K. M. Gamaldo C. E. Salas R. M. E. Ewen J. B. Allen R. P. Sarma S. V. (2018). A novel sleep stage scoring system: combining expert-based rules with a decision tree classi fier, in Proceedings of the Annual International Conference of the IEEE Engineering in Medicine and Biology Society, EMBS (Institute of Electrical and Electronics Engineers Inc.), 3240–3243. 10.1109/EMBC.2018.8513039 PMC6496951 30441082

[B86] Guragain B. Rad A. B. Wang C. Verma A. K. Archer L. Wilson N.. (2019). EEG-based classification of microsleep by means of feature selection: an application in aviation, in Proceedings of the Annual International Conference of the IEEE Engineering in Medicine and Biology Society, EMBS (Honolulu, HI: Institute of Electrical and Electronics Engineers Inc.), 4060–4063. 10.1109/EMBC.2019.8856429 31946764

[B87] Haavisto M. L. Porkka-Heiskanen T. Hublin C. Härmä M. Mutanen P. Müller K.. (2010). Sleep restriction for the duration of a work week impairs multitasking performance. J. Sleep Res. 19, 444–454. 10.1111/j.1365-2869.2010.00823.x 20408942

[B88] Hairston W. D. Whitaker K. W. Ries A. J. Vettel J. M. Bradford J. C. Kerick S. E.. (2014). Usability of four commercially-oriented EEG systems. J. Neural Eng. 11:046018. 10.1088/1741-2560/11/4/046018 24980915

[B89] Hallvig D. Anund A. Fors C. Kecklund G. Åkerstedt T. (2014). Real driving at night - predicting lane departures from physiological and subjective sleepiness. Biol. Psychol. 101, 18–23. 10.1016/j.biopsycho.2014.07.001 25010991

[B90] Han C. H. Kim E. Im C. H. (2020). Development of a brain–computer interface toggle switch with low false-positive rate using respiration-modulated photoplethysmography. Sensors 20:348. 10.3390/s20020348 31936250 PMC7013717

[B91] Hart J. Onceanu D. Sohn C. Wightman D. Vertegaal R. (2009). The attentive hearing aid: eye selection of auditory sources for hearing impaired users, in Lecture Notes in Computer Science (including subseries Lecture Notes in Artificial Intelligence and Lecture Notes in Bioinformatics) (Uppsala; Berlin; Heidelberg: Springer), 19–35. 10.1007/978-3-642-03655-2_4

[B92] Hassan A. R. Bhuiyan M. I. H. (2016). Computer-aided sleep staging using complete ensemble empirical mode decomposition with adaptive noise and bootstrap aggregating. Biomed. Signal Process. Control 24, 1–10. 10.1016/j.bspc.2015.09.002

[B93] He S. Tan H. Li Y. Zhou Y. Yu T. Zhang R.. (2020). EEG- and EOG-based asynchronous hybrid BCI: a system integrating a speller, a web browser, an e-mail client, and a file explorer. IEEE Trans. Neural Syst. Rehabil. Eng. 28, 519–530. 10.1109/TNSRE.2019.2961309 31870987

[B94] He S. Yu T. Gu Z. Li Y. (2017). A hybrid BCI web browser based on EEG and EOG signals, in Proceedings of the Annual International Conference of the IEEE Engineering in Medicine and Biology Society, EMBS (Seogwipo: Institute of Electrical and Electronics Engineers Inc.), 1006–1009. 10.1109/EMBC.2017.8036996 29060044

[B95] He Y. Luu T. P. Nathan K. Nakagome S. Contreras-Vidal J. L. (2018). Data descriptor: a mobile brainbody imaging dataset recorded during treadmill walking with a brain-computer interface. Sci. Data 5, 1–10. 10.1038/sdata.2018.74 29688217 PMC5914288

[B96] Hinrichs H. Scholz M. Baum A. K. Kam J. W. Y. Knight R. T. Heinze H. J. (2020). Comparison between a wireless dry electrode EEG system with a conventional wired wet electrode EEG system for clinical applications. Sci. Rep. 10:5218. 10.1038/s41598-020-62154-0 32251333 PMC7090045

[B97] Hodgson T. L. Ezard G. Hermens F. (2019). Eye movements in neuropsychological tasks, in Current Topics in Behavioral Neurosciences (Springer), 393–418. 10.1007/7854_2019_98 31446573

[B98] Holm A. Lukander K. Korpela J. Sallinen M. Müller K. M. I. (2009). Estimating brain load from the EEG. Sci. World J. 9, 639–651. 10.1100/tsw.2009.83 19618092 PMC5823228

[B99] Hsu W. Y. (2011). Continuous EEG signal analysis for asynchronous BCI application. Int. J. Neural Syst. 21, 335–350. 10.1142/S0129065711002870 21809479

[B100] Hsu W. Y. (2013a). Application of quantum-behaved particle swarm optimization to motor imagery EEG classification. Int. J. Neural Syst. 23:13500263. 10.1142/S0129065713500263 24156669

[B101] Hsu W. Y. (2013b). Independent component analysis and multiresolution asymmetry ratio for brain-computer interface. Clin. EEG Neurosci. 44, 105–111. 10.1177/1550059412463660 23372028

[B102] Hsu W. Y. (2015). Assembling a multi-feature eeg classifier for left-right motor imagery data using wavelet-based fuzzy approximate entropy for improved accuracy. Int. J. Neural Syst. 25:1550037. 10.1142/S0129065715500379 26584583

[B103] Huang Q. Chen Y. Zhang Z. (2019). An EOG-based wheelchair robotic arm system for assisting patients with severe spinal cord injuries. J. Nerual Eng. 16:026021. 10.1088/1741-2552/aafc88 30620927

[B104] Ingre M. Van Leeuwen W. Klemets T. Ullvetter C. Hough S. Kecklund G.. (2014). Validating and extending the three process model of alertness in airline operations. PLoS ONE 9:e0108679. 10.1371/journal.pone.0108679 25329575 PMC4203690

[B105] Issa M. F. Juhasz Z. (2019). Improved EOG artifact removal using wavelet enhanced independent component analysis. Brain Sci. 9:355. 10.3390/brainsci9120355 31817120 PMC6956025

[B106] Itani A. (2009). Health and management in aviation. The experience of air flight pilots and air trafic controllers. Psicol. e Soc. 21, 203–212. 10.1590/S0102-71822009000200007

[B107] Ivorra E. Ortega M. Catalán J. M. Ezquerro S. Lledó L. D. Garcia-Aracil N.. (2018). Intelligent multimodal framework for human assistive robotics based on computer vision algorithms. Sensors 18:2408. 10.3390/s18082408 30042372 PMC6111334

[B108] Jaleel A. Ahmed B. Tafreshi R. Boivin D. B. Streletz L. Haddad N. (2014). Improved spindle detection through intuitive pre-processing of electroencephalogram. J. Neurosci. Methods 233, 1–12. 10.1016/j.jneumeth.2014.05.009 24887741

[B109] Javaid M. A. Weeden J. Flom P. Avitable M. Glazman S. Bodis-Wollner I. (2010). Perisaccadic gamma modulation in Parkinson disease patients and healthy subjects. Clin. EEG Neurosci. 41, 94–101. 10.1177/155005941004100209 20521492

[B110] Javed E. Faye I. Malik A. S. Abdullah J. M. (2017). Removal of BCG artefact from concurrent fMRI-EEG recordings based on EMD and PCA. J. Neurosci. Methods 291, 150–165. 10.1016/j.jneumeth.2017.08.020 28842191

[B111] Jia Y. Tyler C. W. (2019). Measurement of saccadic eye movements by electrooculography for simultaneous EEG recording. Behav. Res. Methods 51, 2139–2151. 10.3758/s13428-019-01280-8 31313136 PMC6797659

[B112] Jiang D. Lu Y. nan Ma Y. Wang Y. (2019). Robust sleep stage classification with single-channel EEG signals using multimodal decomposition and HMM-based refinement. Expert Syst. Appl. 121, 188–203. 10.1016/j.eswa.2018.12.023

[B113] Jiang J. Zhou Z. Yin E. Yu Y. Hu D. (2014). Hybrid brain-computer interface (BCI) based on the EEG and EOG signals, in Bio-Medical Materials and Engineering (Beijing: IOS Press), 2919–2925. 10.3233/BME-141111 25226998

[B114] Justen C. Herbert C. Werner K. Raab M. (2014). Self vs. other: neural correlates underlying agent identification based on unimodal auditory information as revealed by electrotomography (sLORETA). Neuroscience 259, 25–34. 10.1016/j.neuroscience.2013.11.042 24295635

[B115] Kalayci T. Özdamar O. (1995). Wavelet preprocessing for automated neural network detection of EEG spikes. IEEE Eng. Med. Biol. Mag. 14, 160–166. 10.1109/51.376754

[B116] Kang S. Shaikh A. G. (2017). Acquired pendular nystagmus HHS public access. J. Neurol Sci. 375, 8–17. 10.1016/j.jns.2017.01.033 28320194 PMC5363284

[B117] Kanoh S. Ichi-Nohe S. Shioya S. Inoue K. Kawashima R. (2015). Development of an eyewear to measure eye and body movements, in Proceedings of the Annual International Conference of the IEEE Engineering in Medicine and Biology Society (Milan: EMBS), 2267–2270. 10.1109/EMBC.2015.7318844 26736744

[B118] Kaur M. Lagopoulos J. Lee R. S. C. Ward P. B. Naismith S. L. Hickie I. B.. (2013). Longitudinal associations between mismatch negativity and disability in early schizophrenia- and affective-spectrum disorders. Prog. Neuro Psychopharmacol. Biol. Psychiatry 46, 161–169. 10.1016/j.pnpbp.2013.07.002 23851120

[B119] Kempfner J. Jennum P. Nikolic M. Christensen J. A. E. Sorensen H. B. D. (2012). Automatic detection of REM sleep in subjects without atonia, in Proceedings of the Annual International Conference of the IEEE Engineering in Medicine and Biology Society, EMBS (San Diego, CA), 4242–4245. 10.1109/EMBC.2012.6346903 23366864

[B120] Kempfner J. Jennum P. Sorensen H. B. D. Christensen J. A. E. Nikolic M. (2013). Automatic SLEEP staging: from young aduslts to elderly patients using multi-class support vector machine, in Proceedings of the Annual International Conference of the IEEE Engineering in Medicine and Biology Society, EMBS (Osaka: Conf Proc IEEE Eng Med Biol Soc), 5777–5780. 10.1109/EMBC.2013.6610864 24111051

[B121] Kempfner J. Sorensen G. L. Sorensen H. B. D. Jennum P. (2011). Automatic REM sleep detection associated with idiopathic REM sleep behavior disorder, in Proceedings of the Annual International Conference of the IEEE Engineering in Medicine and Biology Society, EMBS (Conf Proc IEEE Eng Med Biol Soc), 6063–6066. 10.1109/IEMBS.2011.6091498 22255722

[B122] Keshavarz B. Berti S. (2014). Integration of sensory information precedes the sensation of vection: a combined behavioral and event-related brain potential (ERP) study. Behav. Brain Res. 259, 131–136. 10.1016/j.bbr.2013.10.045 24211538

[B123] Khalighi S. Sousa T. Nunes U. (2012). Adaptive automatic sleep stage classification under covariate shift, in Proceedings of the Annual International Conference of the IEEE Engineering in Medicine and Biology Society, EMBS (San Diego, CA: Conf Proc IEEE Eng Med Biol Soc), 2259–2262. 10.1109/EMBC.2012.6346412 23366373

[B124] Khalighi S. Sousa T. Oliveira D. Pires G. Nunes U. (2011). Efficient feature selection for sleep staging based on maximal overlap discrete wavelet transform and SVM, in Proceedings of the Annual International Conference of the IEEE Engineering in Medicine and Biology Society, EMBS (Boston, MA), 3306–3309. 10.1109/IEMBS.2011.6090897 22255046

[B125] Khan M. J. Hong K.-S. (2015). Passive BCI based on drowsiness detection: an fNIRS study. Biomed. Opt. Express 6:4063. 10.1364/BOE.6.004063 26504654 PMC4605063

[B126] Khushaba R. N. Kodagoda S. Lal S. Dissanayake G. (2011). Driver drowsiness classification using fuzzy wavelet-packet-based feature-extraction algorithm. IEEE Trans. Biomed. Eng. 58, 121–131. 10.1109/TBME.2010.2077291 20858575

[B127] Killane I. Browett G. Reilly R. B. (2013). Measurement of attention during movement: acquisition of ambulatory EEG and cognitive performance from healthy young adults, in Proceedings of the Annual International Conference of the IEEE Engineering in Medicine and Biology Society, EMBS (Osaka: Conf Proc IEEE Eng Med Biol Soc), 6397–6400. 10.1109/EMBC.2013.6611018 24111205

[B128] Kim D. J. Bolbecker A. R. Howell J. Rass O. Sporns O. Hetrick W. P.. (2013). Disturbed resting state EEG synchronization in bipolar disorder: a graph-theoretic analysis. Neuroimage Clin. 2, 414–423. 10.1016/j.nicl.2013.03.007 24179795 PMC3777715

[B129] Kirenskaya A. V. Myamlin V. V. Novototsky-Vlasov V. Y. Pletnikov M. V. Kozlovskaya I. B. (2011). The contingent negative variation laterality and dynamics in antisaccade task in normal and unmedicated schizophrenic subjects. Span. J. Psychol. 14, 869–883. 10.5209/rev_SJOP.2011.v14.n2.34 22059332

[B130] Kleifges K. Bigdely-Shamlo N. Kerick S. E. Robbins K. A. (2017). BLINKER: automated extraction of ocular indices from EEG enabling large-scale analysis. Front. Neurosci. 11:12. 10.3389/fnins.2017.00012 28217081 PMC5289990

[B131] Klein A. Skrandies W. (2013). A reliable statistical method to detect eyeblink-artefacts from electroencephalogram data only. Brain Topogr. 26, 558–568. 10.1007/s10548-013-0281-2 23532464

[B132] Klimesch W. Sauseng P. Hanslmayr S. (2007). EEG alpha oscillations: the inhibition-timing hypothesis. Brain Res. Rev. 53, 63–88. 10.1016/j.brainresrev.2006.06.003 16887192

[B133] Klimesch W. Vogt F. Doppelmayr M. (1999). Interindividual differences in alpha and theta power reflect memory performance. Intelligence 27, 347–362. 10.1016/S0160-2896(99)00027-610209231

[B134] Klok A. B. Edin J. Cesari M. Olesen A. N. Jennum P. Sorensen H. B. D. (2018). A new fully automated random-forest algorithm for sleep staging, in Proceedings of the Annual International Conference of the IEEE Engineering in Medicine and Biology Society, EMBS (Honolulu, HI: Institute of Electrical and Electronics Engineers Inc.), 4920–4923. 10.1109/EMBC.2018.8513413 30441446

[B135] Koch H. Christensen J. A. E. Frandsen R. Zoetmulder M. Arvastson L. Christensen S. R.. (2014). Automatic sleep classification using a data-driven topic model reveals latent sleep states. J. Neurosci. Methods 235, 130–137. 10.1016/j.jneumeth.2014.07.002 25016288

[B136] Koch H. Jennum P. Christensen J. A. E. (2019). Automatic sleep classification using adaptive segmentation reveals an increased number of rapid eye movement sleep transitions. J. Sleep Res. 28:e12780. 10.1111/jsr.12780 30346084

[B137] Kong D. Soon C. (2012). Functional imaging correlates of impaired distractor suppression following sleep deprivation. Neuroimage 61, 50–55. 10.1016/j.neuroimage.2012.02.081 22426349

[B138] Kong W. Zhou Z. Hu S. Zhang J. Babiloni F. Dai G. (2013). Automatic and direct identification of blink components from scalp EEG. Sensors 13, 10783–10801. 10.3390/s130810783 23959240 PMC3812628

[B139] Korkalainen H. Leppanen T. Aakko J. Nikkonen S. Kainulainen S. Leino A.. (2019). Accurate deep learning-based sleep staging in a clinical population with suspected obstructive sleep apnea. IEEE J. Biomed. Heal. Inform. 24, 2073–2081. 10.1109/JBHI.2019.2951346 31869808

[B140] Krakovská A. Mezeiová K. (2011). Automatic sleep scoring: a search for an optimal combination of measures. Artif. Intell. Med. 53, 25–33. 10.1016/j.artmed.2011.06.004 21742473

[B141] Krauss P. Schilling A. Bauer J. Tziridis K. Metzner C. Schulze H.. (2018). Analysis of multichannel EEG patterns during human sleep: a novel approach. Front. Hum. Neurosci. 12:121. 10.3389/fnhum.2018.00121 29636673 PMC5880946

[B142] Kumar D. Das A. Lahiri U. Dutta A. (2016). A human-machine-interface integrating low-cost sensors with a neuromuscular electrical stimulation system for post-stroke balance rehabilitation. J. Vis. Exp. 2016:52394. 10.3791/52394 PMC4941915 27166666

[B143] Kuo T. B. J. Chen C. Y. Hsu Y. C. Yang C. C. H. (2016). EEG beta power and heart rate variability describe the association between cortical and autonomic arousals across sleep. Auton. Neurosci. Basic Clin. 194, 32–37. 10.1016/j.autneu.2015.12.001 26681575

[B144] Laszlo S. Ruiz-Blondet M. Khalifian N. Chu F. Jin Z. (2014). A direct comparison of active and passive amplification electrodes in the same amplifier system. J. Neurosci. Methods 235, 298–307. 10.1016/j.jneumeth.2014.05.012 25075801

[B145] Lee M. H. Williamson J. Won D. O. Fazli S. Lee S. W. (2018). A high performance spelling system based on EEG-EOG signals with visual feedback. IEEE Trans. Neural Syst. Rehabil. Eng. 26, 1443–1459. 10.1109/TNSRE.2018.2839116 29985154

[B146] Lee S. H. Sung K. Lee K. S. Moon E. Kim C. G. (2014). Mismatch negativity is a stronger indicator of functional outcomes than neurocognition or theory of mind in patients with schizophrenia. Prog. Neuro Psychopharmacol. Biol. Psychiatry 48, 213–219. 10.1016/j.pnpbp.2013.10.010 24161665

[B148] Li T. Zhang J. Xue T. Wang B. (2017). Development of a novel motor imagery control technique and application in a gaming environment. Comput. Intell. Neurosci. 2017:5863512. 10.1155/2017/5863512 28572817 PMC5441123

[B149] Li Y. Yingle F. Gu L. Qinye T. (2009). Sleep stage classification based on EEG hilbert-huang transform, in 2009 4th IEEE Conference on Industrial Electronics and Applications, ICIEA (Xi'an) 3676–3681. 10.1109/ICIEA.2009.5138842

[B150] Liang S. F. Kuo C. E. Hu Y. H. Cheng Y. S. (2011). A rule-based automatic sleep staging method, in Proceedings of the Annual International Conference of the IEEE Engineering in Medicine and Biology Society, EMBS (Boston, MA: Conf Proc IEEE Eng Med Biol Soc), 6067–6070. 10.1109/IEMBS.2011.6091499 22255723

[B151] Liao L. De Chen C. Y. Wang I. J. Chen S. F. Li S. Y. Chen B. W.. (2012). Gaming control using a wearable and wireless EEG-based brain-computer interface device with novel dry foam-based sensors. J. Neuroeng. Rehabil. 9, 1–12. 10.1186/1743-0003-9-5 22284235 PMC3283495

[B152] Lisberger S. G. Westbrook L. E. (1985). Properties of visual inputs that initiate horizontal smooth pursuit eye movements in monkeys. J. Neurosci. 5, 1662–1673. 10.1523/JNEUROSCI.05-06-01662.1985 4009252 PMC6565252

[B153] Liu R. Zhang Z. Duan F. Zhou X. Meng Z. (2017). Identification of anisomerous motor imagery EEG signals based on complex algorithms. Comput. Intell. Neurosci. 2017:2727856. 10.1155/2017/2727856 28874909 PMC5569879

[B154] Liu Y. Zhou Z. Hu D. (2011). Gaze independent brain-computer speller with covert visual search tasks. Clin. Neurophysiol. 122, 1127–1136. 10.1016/j.clinph.2010.10.049 21163695

[B155] Looney D. Park C. Kidmose P. Rank M. L. Ungstrup M. Rosenkranz K.. (2011). An in-the-ear platform for recording electroencephalogram, in Proceedings of the Annual International Conference of the IEEE Engineering in Medicine and Biology Society, EMBS (Boston, MA), 6882–6885. 10.1109/IEMBS.2011.6091733 22255920

[B156] Ma J. Bayram S. Tao P. Svetnik V. (2011). High-throughput ocular artifact reduction in multichannel electroencephalography (EEG) using component subspace projection. J. Neurosci. Methods 196, 131–140. 10.1016/j.jneumeth.2011.01.007 21236300

[B157] Ma J. Zhang Y. Cichocki A. Matsuno F. (2015). A novel EOG/EEG hybrid human-machine interface adopting eye movements and ERPs: application to robot control. IEEE Trans. Biomed. Eng. 62, 876–889. 10.1109/TBME.2014.2369483 25398172

[B158] Ma J. X. Shi L. C. Lu B. L. (2010). Vigilance estimation by using electrooculographic features, in 2010 Annual International Conference of the IEEE Engineering in Medicine and Biology Society, EMBC'10 (Buenos Aires), 6591–6594. 10.1109/IEMBS.2010.5627122 21096514

[B159] MacDonald B. Barry R. J. (2014). Trial effects in single-trial ERP components and autonomic responses at very long ISIs. Int. J. Psychophysiol. 92, 99–112. 10.1016/j.ijpsycho.2014.03.007 24681245

[B160] Manabe H. Fukumoto M. Yagi T. (2013). Automatic drift calibration for EOG-based gaze input interface, in Proceedings of the Annual International Conference of the IEEE Engineering in Medicine and Biology Society, EMBS (Osaka), 53–56. 10.1109/EMBC.2013.6609435 24109622

[B161] Meyberg S. Sommer W. Dimigen O. (2017). How microsaccades relate to lateralized ERP components of spatial attention: a co-registration study. Neuropsychologia 99, 64–80. 10.1016/j.neuropsychologia.2017.02.023 28254651

[B162] Min B. K. Dähne S. Ahn M. H. Noh Y. K. Müller K. R. (2016). Decoding of top-down cognitive processing for SSVEP-controlled BMI. Sci. Rep. 6:36567. 10.1038/srep36267 27808125 PMC5093690

[B163] Mirkovic B. Bleichner M. G. De Vos M. Debener S. (2016). Target speaker detection with concealed EEG around the ear. Front. Neurosci. 10:349. 10.3389/fnins.2016.00349 27512364 PMC4961688

[B164] Müller C. Nicoletti C. Omlin S. Brink M. Läubli T. (2015). Relationship between sleep stages and nocturnal trapezius muscle activity. J. Electromyogr. Kinesiol. 25, 457–462. 10.1016/j.jelekin.2015.01.010 25765124

[B165] Nguyen T. Ahn S. Jang H. Jun S. C. Kim J. G. (2017). Utilization of a combined EEG/NIRS system to predict driver drowsiness. Sci. Rep. 7:43933. 10.1038/srep43933 28266633 PMC5339693

[B166] Nguyen T. Babawale O. Kim T. Jo H. J. Liu H. Kim J. G. (2018). Exploring brain functional connectivity in rest and sleep states: a fNIRS study. Sci. Rep. 8:16144. 10.1038/s41598-018-33439-2 30385843 PMC6212555

[B167] Niemenlehto P. H. (2009). Constant false alarm rate detection of saccadic eye movements in electro-oculography. Comput. Methods Programs Biomed. 96, 158–171. 10.1016/j.cmpb.2009.04.011 19482371

[B168] Nogueira W. Dolhopiatenko H. Schierholz I. Büchner A. Mirkovic B. Bleichner M. G.. (2019). Decoding selective attention in normal hearing listeners and bilateral cochlear implant users with concealed ear EEG. Front. Neurosci. 13:720. 10.3389/fnins.2019.00720 31379479 PMC6657402

[B169] Nolan H. Whelan R. Reilly R. B. (2010). FASTER: fully automated statistical thresholding for EEG artifact rejection. J. Neurosci. Methods 192, 152–162. 10.1016/j.jneumeth.2010.07.015 20654646

[B170] Noureddin B. Lawrence P. D. Birch G. E. (2012). Online removal of eye movement and blink EEG artifacts using a high-speed eye tracker. IEEE Trans. Biomed. Eng. 59, 2103–2110. 10.1109/TBME.2011.2108295 21278013

[B171] Oken B. S. Salinsky M. C. Elsas S. M. (2006). Vigilance, alertness, or sustained attention: physiological basis and measurement. Clin. Neurophysiol. 117, 1885–1901. 10.1016/j.clinph.2006.01.017 16581292 PMC2865224

[B172] Olsen A. V. Stephansen J. Leary E. Peppard P. E. Sheungshul H. Jenum P.. (2017). Diagnostic value of sleep stage dissociation as visualized on a 2-dimensional sleep state space in human narcolepsy. J. Neurosci. Methods 282, 9–19. 10.1016/j.jneumeth.2017.02.004 28219726

[B173] Pan S. T. Kuo C. E. Zeng J. H. Liang S. F. (2012). A transition-constrained discrete hidden Markov model for automatic sleep staging. Biomed. Eng. Online. 11:52. 10.1186/1475-925X-11-52 22908930 PMC3462123

[B174] Parasuraman R. Jiang Y. (2012). Individual differences in cognition, affect, and performance: Behavioral, neuroimaging, and molecular genetic approaches. Neuroimage 59, 70–82. 10.1016/j.neuroimage.2011.04.040 21569853 PMC3482491

[B175] Petrie K. J. Dawson A. G. (1997). Symptoms of fatigue and coping strategies in international pilots. Int. J. Aviat. Psychol. 7, 251–258. 10.1207/s15327108ijap0703_5

[B176] Pettersson K. Jagadeesan S. Lukander K. Henelius A. Hæggström E. Müller K. (2013). Algorithm for automatic analysis of electro-oculographic data. Biomed. Eng. Online 12:110. 10.1186/1475-925X-12-110 24160372 PMC3830504

[B177] Peysakhovich V. Dehais F. Duchowski A. T. (2019). Why is eye tracking an essential part of neuroergonomics? in Neuroergonomics: The Brain at Work and in Everyday Life (Elsevier), 27–30. 10.1016/B978-0-12-811926-6.00004-X

[B178] Peysakhovich V. Lefrançois O. Dehais F. Causse M. (2018). The neuroergonomics of aircraft cockpits: the four stages of eye-tracking integration to enhance flight safety. Safety 4:8. 10.3390/safety4010008

[B179] Pham T. T. H. Croft R. J. Cadusch P. J. (2011a). Temporal stability of regression-based electrooculographic correction coefficients. Psychophysiology 48, 96–101. 10.1111/j.1469-8986.2010.01036.x 20536903

[B180] Pham T. T. H. Croft R. J. Cadusch P. J. Barry R. J. (2011b). A test of four EOG correction methods using an improved validation technique. Int. J. Psychophysiol. 79, 203–210. 10.1016/j.ijpsycho.2010.10.008 21034784

[B181] Postma M. A. Schellekens J. M. Hanson E. K. Hoogeboom P. J. (2005). Fz Theta Divided by Pz Alpha as an Index of Task Load During a PC-Based Air Traffic Control Simulation. Maastricht: Shaker Publishing, 465–469.

[B182] Punsawad Y. Wongsawat Y. Parnichkun M. (2010). Hybrid EEG-EOG brain-computer interface system for practical machine control, in 2010 Annual International Conference of the IEEE Engineering in Medicine and Biology Society, EMBC'10 (Buenos Aires), 1360–1363. 10.1109/IEMBS.2010.5626745 21096331

[B183] Radha M. Garcia-Molina G. Poel M. Tononi G. (2014). Comparison of feature and classifier algorithms for online automatic sleep staging based on a single EEG signal, in 2014 36th Annual International Conference of the IEEE Engineering in Medicine and Biology Society, EMBC (Chicago, IL), 1876–1880. 10.1109/EMBC.2014.6943976 25570344

[B184] Rajesh N. (2014). EOG controlled motorized wheelchair for disabled persons. Int. J. Med. Heal. Biomed. Bioeng. Pharm. Eng. 8, 302–305. 10.5281/zenodo.1337385

[B185] Rao R. Derakhshani R. (2005). A comparison of EEG preprocessing methods using time delay neural networks, in 2nd International IEEE EMBS Conference on Neural Engineering (Arlington, VA), 262–264.

[B186] Reed C. M. Birch K. G. Kamiński J. Sullivan S. Chung J. M. Mamelak A. N.. (2017). Automatic detection of periods of slow wave sleep based on intracranial depth electrode recordings. J. Neurosci. Methods 282, 1–8. 10.1016/j.jneumeth.2017.02.009 28238858 PMC5455770

[B187] Reis P. M. R. Hebenstreit F. Gabsteiger F. von Tscharner V. Lochmann M. (2014). Methodological aspects of EEG and body dynamics measurements during motion. Front. Hum. Neurosci. 8:156. 10.3389/fnhum.2014.00156 24715858 PMC3970018

[B188] Rezaei M. Mohammadi H. Khazaie H. (2017). EEG/EOG/EMG data from a cross sectional study on psychophysiological insomnia and normal sleep subjects. Data Br. 15, 314–319. 10.1016/j.dib.2017.09.033 29214192 PMC5712051

[B189] Rosales-Lagarde A. Rodriguez-Torres E. E. Itzá-Ortiz B. A. Miramontes P. Vázquez-Tagle G. Enciso-Alva J. C.. (2018). The color of noise and weak stationarity at the NREM to REM sleep transition in mild cognitive impaired subjects. Front. Psychol. 9:1205. 10.3389/fpsyg.2018.01205 30065684 PMC6056768

[B190] Roy R. N. Frey J. (2016). Neurophysiological Markers for Passive Brain-Computer Interfaces, in Brain-Computer Interfaces 1: Foundations and Methods (Wiley Online Library), 85–100. 10.1002/9781119144977.ch5

[B191] Sameni R. Gouy-Pailler C. (2014). An iterative subspace denoising algorithm for removing electroencephalogram ocular artifacts. J. Neurosci. Methods 225, 97–105. 10.1016/j.jneumeth.2014.01.024 24486874

[B192] Sauvet F. Bougard C. Coroenne M. Lely L. Van Beers P. Elbaz M.. (2014). In-flight automatic detection of vigilance states using a single EEG channel. IEEE Trans. Biomed. Eng. 61, 2840–2847. 10.1109/TBME.2014.2331189 24967979

[B193] Scarlatelli-Lima A. V. Sukys-Claudino L. Watanabe N. Guarnieri R. Walz R. Lin K. (2016). How do people with drug-resistant mesial temporal lobe epilepsy sleep? A clinical and video-EEG with EOG and submental EMG for sleep staging study. eNeurologicalSci 4, 34–41. 10.1016/j.ensci.2016.06.002 29430547 PMC5803108

[B194] Semmlow J. L. Yuan W. Alvarez T. L. (1998). Evidence for separate control of slow version and vergence eye movements: support for hering's law. Vision Res. 38, 1145–1152. 10.1016/S0042-6989(97)00251-4 9666973

[B195] Shustak S. Inzelberg L. Steinberg S. Rand D. David Pur M. Hillel I.. (2018). Home monitoring of sleep with a temporary-tattoo EEG, EOG and EMG electrode array: a feasibility study. J. Neural Eng. 16:026024. 10.1088/1741-2552/aafa05 30566912

[B196] Singh H. Singh J. (2012). Human eye tracking and related issues: a review. Int. J. Sci. Res. Publ. 2.

[B197] Singh S. Shukla G. Goyal V. Srivastava A. K. Singh M. B. Vibha D.. (2014). Impact of sleep on the localizing value of video EEG in patients with refractory focal seizures - a prospective video-EEG with EOG and submental EMG study. Clin. Neurophysiol. 125, 2337–2343. 10.1016/j.clinph.2014.03.021 24856459

[B198] Skotte J. H. Nøjgaard J. K. Jørgensen L. V. Christensen K. B. Sjøgaard G. (2007). Eye blink frequency during different computer tasks quantified by electrooculography. Eur. J. Appl. Physiol. 99, 113–119. 10.1007/s00421-006-0322-6 17115181

[B199] Smith-Jentsch K. A. Brannick M. T. Salas E. (2001). To transfer or not to transfer? Investigating the combined effects of trainee characteristics, team leader support, and team climate. J. Appl. Psychol. 86, 279–292. 10.1037/0021-9010.86.2.279 11393440

[B200] Soekadar S. R. Witkowski M. Vitiello N. Birbaumer N. (2015). An EEG/EOG-based hybrid brain-neural computer interaction (BNCI) system to control an exoskeleton for the paralyzed hand. Biomed. Tech. 60, 199–205. 10.1515/bmt-2014-0126 25490027

[B201] Sokolovsky M. Guerrero F. Paisarnsrisomsuk S. Ruiz C. Alvarez S. A. (2019). Deep learning for automated feature discovery and classification of sleep stages. IEEE/ACM Trans. Comput. Biol. Bioinforma. 1545–5963. 10.1109/TCBB.2019.2912955 31027049

[B202] Sommer D. Golz M. (2010). Evaluation of PERCLOS based current fatigue monitoring technologies, in 2010 Annual International Conference of the IEEE Engineering in Medicine and Biology Society, EMBC'10, (Buenos Aires) 4456–4459. 10.1109/IEMBS.2010.5625960 21095770

[B203] Stochholm A. Mikkelsen K. Kidmose P. (2016). Automatic sleep stage classification using ear-EEG, in Proceedings of the Annual International Conference of the IEEE Engineering in Medicine and Biology Society, EMBS (Orlando, FL: Institute of Electrical and Electronics Engineers Inc.), 4751–4754. 10.1109/EMBC.2016.7591789 28269332

[B204] Sun C. Chen C. Fan J. Li W. Zhang Y. Chen W. (2019). A hierarchical sequential neural network with feature fusion for sleep staging based on EOG and RR signals - IOPscience. J. Neural Eng. 16:066020. 10.1088/1741-2552/ab39ca 31394522

[B205] Supratak A. Dong H. Wu C. Guo Y. (2017). DeepSleepNet: a model for automatic sleep stage scoring based on raw single-channel EEG. IEEE Trans. Neural Syst. Rehabil. Eng. 25, 1998–2008. 10.1109/TNSRE.2017.2721116 28678710

[B206] Svensson E. Angelborg-Thanderez M. Sjöberg L. Olsson S. (1997). Information complexity-mental workload and performance in combat aircraft. Ergonomics 40, 362–380. 10.1080/001401397188206 11536799

[B207] Tagluk M. E. Sezgin N. Akin M. (2010). Estimation of sleep stages by an artificial neural network employing EEG, EMG and EOG. J. Med. Syst. 34, 717–725. 10.1007/s10916-009-9286-5 20703927

[B208] Tan T. Hakenberg J. P. Guan C. (2013). Estimation of glance from EEG for cursor control. Annu. Int. Conf. IEEE Eng. Med. Biol. Soc. 2013, 2919–2923. 10.1109/EMBC.2013.6610151 24110338

[B209] Tang Y. Zhang X. Simmonite M. Li H. Zhang T. Guo Q.. (2013). Hyperactivity within an extensive cortical distribution associated with excessive sensitivity in error processing in unmedicated depression: a combined event-related potential and sLORETA study. Int. J. Psychophysiol. 90, 282–289. 10.1016/j.ijpsycho.2013.09.001 24056021

[B210] Toivanen M. Pettersson K. Lukander K. (2015). A probabilistic real-time algorithm for detecting blinks, saccades, and fixations from EOG data. J. Eye Mov. Res. 8, 1–14. 10.16910/jemr.8.2.1

[B211] Torres-Valencia C. A. Alvarez M. A. Orozco-Gutierrez A. A. (2014). Multiple-output support vector machine regression with feature selection for arousal/valence space emotion assessment, in 2014 36th Annual International Conference of the IEEE Engineering in Medicine and Biology Society EMBC (Chicago, IL: IEEE), 970–973. 10.1109/EMBC.2014.6943754 25570122

[B212] Tyagi A. Nehra V. (2018). A Comparison of Feature Extraction and Dimensionality Reduction Techniques for EEG-Based BCI System. IUP J. Comput. Sci. 11, 51–66. Available online at: https://ssrn.com/abstract=3159745

[B213] Usakli A. Gurkan S. Aloise F. Vecchiato G. Babiloni F. (2010). On the use of electrooculogram for efficient human computer interfaces. Comput. Intell. Neurosci. 2010:135629. 10.1155/2010/135629 19841687 PMC2763213

[B214] Velazquez J. (2018). The presence of behavioral traps in U.S. airline accidents: a qualitative analysis. Safety 4:2. 10.3390/safety4010002

[B215] Verdière K. J. Roy R. N. Dehais F. (2018). Detecting pilot's engagement using fNIRS connectivity features in an automated vs. manual landing scenario. Front. Hum. Neurosci. 12:6. 10.3389/fnhum.2018.00006 29422841 PMC5788966

[B216] Verma G. K. Tiwary U. S. (2014). Multimodal fusion framework: a multiresolution approach for emotion classification and recognition from physiological signals. Neuroimage 102, 162–172. 10.1016/j.neuroimage.2013.11.007 24269801

[B217] Von Rosenberg W. Chanwimalueang T. Goverdovsky V. Looney D. Sharp D. Mandic D. P. (2016). Smart helmet:wearable multichannel ECG and EEG. IEEE J. Transl. Eng. Heal. Med. 4:2700111. 10.1109/JTEHM.2016.2609927 PMC5127696 27957405

[B218] Wang D. Miao D. Blohm G. (2012). Multi-class motor imagery EEG decoding for brain-computer interfaces. Front. Neurosci. 6:151. 10.3389/fnins.2012.00151 23087607 PMC3466781

[B219] Wang F. Xu Q. Fu R. (2019). Study on the effect of man-machine response mode to relieve driving fatigue based on EEG and EOG. Sensors 19:4883. 10.3390/s19224883 31717422 PMC6891316

[B220] Wang G. Teng C. Li K. Zhang Z. Yan X. (2016). The removal of EOG artifacts from EEG signals using independent component analysis and multivariate empirical mode decomposition. IEEE J. Biomed. Heal. Informatics 20, 1301–1308. 10.1109/JBHI.2015.2450196 26126290

[B221] Wang H. Li Y. Long J. Yu T. Gu Z. (2014a). An asynchronous wheelchair control by hybrid EEG–EOG brain–computer interface. Cogn. Neurodyn. 8, 399–409. 10.1007/s11571-014-9296-y 25206933 PMC4155067

[B222] Wang X. W. Nie D. Lu B. L. (2014b). Emotional state classification from EEG data using machine learning approach. Neurocomputing 129, 94–106. 10.1016/j.neucom.2013.06.046

[B223] Whitehead K. Laudiano-Dray M. P. Meek J. Lorenzo F. (2018). Emergence of mature cortical activity in wakefulness and sleep in healthy preterm and full-term infants. Sleep 41:zsy096. 10.1093/sleep/zsy096 29762768 PMC6093466

[B224] Wilson N. Guragain B. Verma A. Archer L. Tavakolian K. (2020). Blending human and machine: feasibility of measuring fatigue through the aviation headset. Hum. Factors. 62, 553–564. 10.1177/0018720819849783 31180741

[B225] Winkler I. Debener S. Muller K. R. Tangermann M. (2015). On the influence of high-pass filtering on ICA-based artifact reduction in EEG-ERP. Annu. Int. Conf. IEEE Eng. Med. Biol. Soc. 2015, 4101–4105. 10.1109/EMBC.2015.7319296 26737196

[B226] Witkowski M. Cortese M. Cempini M. Mellinger J. Vitiello N. Soekadar S. R. (2014). Enhancing brain-machine interface (BMI) control of a hand exoskeleton using electrooculography (EOG). J. Neuroeng. Rehabil. 11:165. 10.1186/1743-0003-11-165 25510922 PMC4274709

[B227] Wright N. McGown A. (2001). Vigilance on the civil flight deck: Incidence of sleepiness and sleep during long-haul flights and associated changes in physiological parameters. Ergonomics 44, 82–106. 10.1080/00140130150203893 11214900

[B228] Wu J. Zhang J. Ding X. Li R. Zhou C. (2013). The effects of music on brain functional networks: a network analysis. Neuroscience 250, 49–59. 10.1016/j.neuroscience.2013.06.021 23806719

[B229] Yaghouby F. Sunderam S. (2015). Quasi-supervised scoring of human sleep in polysomnograms using augmented input variables. Comput. Biol. Med. 59, 54–63. 10.1016/j.compbiomed.2015.01.012 25679475 PMC4447106

[B230] Yamada F. (1998). Frontal midline theta rhythm and eyeblinking activity during a VDT task and a video game: useful tools for psychophysiology in ergonomics. Ergonomics 41, 678–688. 10.1080/001401398186847 9613228

[B231] Yamagishi K. Hori J. Miyakawa M. (2006). Development of EOG-based communication system controlled by eight-directional eye movements. Conf. Proc. IEEE Eng. Med. Biol. Soc. 2006, 2574–2577. 10.1109/IEMBS.2006.259914 17945724

[B232] Yamaguchi K. Hoshiyama M. Takano M. (2011). Biological observation during the daytime of elderly patients with advanced dementia cared for with and without artificial nutrition by percutaneous endoscopic gastrostomy. Geriatr. Gerontol. Int. 11, 221–228. 10.1111/j.1447-0594.2010.00657.x 21050349

[B233] Yan M. Tamura H. Tanno K. (2013). Gaze estimation using electrooculogram signals and its mathematical modeling, in Proceedings of The International Symposium on Multiple-Valued Logic (Toyama), 18–22.

[B234] Yildirim O. Baloglu U. B. Acharya U. R. (2019). A deep learning model for automated sleep stages classification using PSG signals. Int. J. Environ. Res. Public Health 16:599. 10.3390/ijerph16040599 30791379 PMC6406978

[B235] Yu Y. Liu Y. Yin E. Jiang J. Zhou Z. Hu D. (2019). An asynchronous hybrid spelling approach based on EEG-EOG signals for chinese character input. IEEE Trans. Neural Syst. Rehabil. Eng. 27, 1292–1302. 10.1109/TNSRE.2019.2914916 31071045

[B236] Zander T. O. Kothe C. Jatzev S. Gaertner M. (2010). Enhancing Human-Computer Interaction with Input from Active and Passive Brain-Computer Interfaces, in Brain-Computer Interfaces. Human-Computer Interaction Series, eds Tan D. Nijholt A. (London: Springer), 181–199. 10.1007/978-1-84996-272-8_11

[B237] Zeng H. Song A. (2014). Removal of EOG artifacts from EEG recordings using stationary subspace analysis. Sci. World J. 2014:259121. 10.1155/2014/259121 24550696 PMC3914441

[B238] Zennifa F. Ageno S. Hatano S. Iramina K. (2018). Hybrid system for engagement recognition during cognitive tasks using a CFS + KNN algorithm. Sensors 18:3691. 10.3390/s18113691 30380784 PMC6263401

[B239] Zhang C. Yu X. L. Rao N. N. (2019a). Linear descriptor parameter analysis of mental fatigue's EEG in multi-task. J. Univ. Electron. Sci. Technol. China 48, 613–618. 10.3969/j.issn.1001-0548.2019.04.020

[B240] Zhang J. Wang B. Zhang C. Xiao Y. Wang M. Y. (2019b). An EEG/EMG/EOG-based multimodal human-machine interface to real-time control of a soft robot hand. Front. Neurorobot. 13:7. 10.3389/fnbot.2019.00007 30983986 PMC6449448

[B241] Zhang L. Chi Y. M. Edelstein E. Schulze J. Gramann K. Velasquez A.. (2010a). Wireless physiological monitoring and ocular tracking: 3D calibration in a fully-immersive virtual health care environment, in 2010 Annual International Conference of the IEEE Engineering in Medicine and Biology Society EMBC'10 (Buenos Aires: IEEE Computer Society), 4464–4467. 10.1109/IEMBS.2010.5625969 21095772

[B242] Zhang L. He W. He C. Wang P. (2010b). Improving mental task classification by adding high frequency band information. J. Med. Syst. 34, 51–60. 10.1007/s10916-008-9215-z 20192055

[B243] Zhang Y. Zhang X. Liu W. Luo Y. Yu E. Zou K.. (2014). Automatic sleep staging using multi-dimensional feature extraction and multi-kernel fuzzy support vector machine. J. Healthc. Eng. 5, 505–520. 10.1260/2040-2295.5.4.505 25516130

[B244] Zheng W.-L. Lu B.-L. (2016). A multimodal approach to estimating vigilance using EEG and forehead EOG - IOPscience. J. Neural Eng. 14:026017. 10.1088/1741-2552/aa5a98 28102833

[B245] Zhimin Z. Shoushui W. Guohun Z. Feifei L. Yuwen L. Xiaotong D.. (2018). Efficient sleep classification based on entropy features and a support vector machine classifier - IOPscience. Physiol. Meas. 39:1–6. 10.1088/1361-6579/aae943 30475743

[B246] Zhou Y. He S. Huang Q. Li Y. (2020). A hybrid asynchronous brain-computer interface combining SSVEP and EOG Signals. IEEE Trans. Biomed. Eng. 67, 2881–2892. 10.1109/TBME.2020.2972747 32070938

[B247] Zibrandtsen I. Kidmose P. Otto M. Ibsen J. Kjaer T. W. (2016). Case comparison of sleep features from ear-EEG and scalp-EEG. Sleep Sci. 9, 69–72. 10.1016/j.slsci.2016.05.006 27656268 PMC5021956

